# Shared and Distinct Features of Human Milk and Infant Stool Viromes

**DOI:** 10.3389/fmicb.2018.01162

**Published:** 2018-06-01

**Authors:** Pia S. Pannaraj, Melissa Ly, Chiara Cerini, Monica Saavedra, Grace M. Aldrovandi, Abdul A. Saboory, Kevin M. Johnson, David T. Pride

**Affiliations:** ^1^Division of Infectious Diseases, Children’s Hospital Los Angeles, Los Angeles, CA, United States; ^2^Department of Pediatrics, Molecular Microbiology and Immunology, Keck School of Medicine, University of Southern California, Los Angeles, CA, United States; ^3^Department of Pathology, University of California, San Diego, San Diego, CA, United States; ^4^Department of Pediatrics, University of California, Los Angeles, Los Angeles, CA, United States; ^5^Department of Medicine, University of California, San Diego, San Diego, CA, United States

**Keywords:** human microbiome, virome, virobiota, breast milk, milk microbiota

## Abstract

Infants acquire many of their microbes from their mothers during the birth process. The acquisition of these microbes is believed to be critical in the development of the infant immune system. Bacteria also are transmitted to the infant through breastfeeding, and help to form the microbiome of the infant gastrointestinal (GI) tract; it is unknown whether viruses in human milk serve to establish an infant GI virome. We examined the virome contents of milk and infant stool in a cohort of mother-infant pairs to discern whether milk viruses colonize the infant GI tract. We observed greater viral alpha diversity in milk than in infant stool, similar to the trend we found for bacterial communities from both sites. When comparing beta diversity, viral communities were mostly distinguishable between milk and infant stool, but each was quite distinct from adult stool, urine, and salivary viromes. There were significant differences in viral families in the infant stool (abundant bacteriophages from the family Siphoviridae) compared to milk (abundant bacteriophages from the family Myoviridae), which may reflect significant differences in the bacterial families identified from both sites. Despite the differences in viral taxonomy, we identified a significant number of shared viruses in the milk and stool from all mother-infant pairs. Because of the significant proportion of bacteriophages transmitted in these mother-infant pairs, we believe the transmission of milk phages to the infant GI tract may help to shape the infant GI microbiome.

## Introduction

The human body carries communities of microbes that inhabit virtually every available niche on both its inner and outer surfaces (Ding and Schloss, 2014). Most studies to date have focused on bacteria that inhabit body surfaces; however, a growing number of studies suggest that viruses also play an important role in health and disease (Abeles et al., 2015; Reyes et al., 2015; Columpsi et al., 2016; Ly et al., 2016; Duranti et al., 2017; Wylie, 2017). Studies of viruses inhabiting human body surfaces have generally suffered from a strong focus on DNA rather than RNA viruses, biases toward characterization of non-enveloped viruses, specimen preservation techniques that may reduce representation of certain viruses, and a relative lack of quantitative techniques. Despite these limitations, human virome studies have clearly demonstrated that viruses inhabit all the same niches as those inhabited by bacteria. For example, viruses inhabit the mouth (Abeles et al., 2014; Ly et al., 2014), respiratory tract (Wylie, 2017), gastrointestinal (GI) tract (Columpsi et al., 2016), bladder (Santiago-Rodriguez et al., 2015a), skin (Hannigan et al., 2015), and even the blood (Sauvage et al., 2016) of healthy individuals. The significant representation of viruses on the human body surfaces that have been studied to date suggests that there are no parts of the human body free of viruses.

The human microbiome plays a significant role in human health and disease, including its potential role in immune maturation (Gensollen et al., 2016; Laforest-Lapointe and Arrieta, 2017), direct pathogenicity (Eloe-Fadrosh and Rasko, 2013; Sanz et al., 2015), and in protection against pathogen invasion (Edelblum et al., 2017). Studies of the virome in early life are limited (Breitbart et al., 2008; Lim et al., 2015; Duranti et al., 2017). Increasing attention has recently been focused on the extent to which the microbiome may be shared amongst close contacts, so that the microbiome of an individual may have characteristics that are relatively individual-specific, but that individuals have the capacity to share their microbiota with their environment (Lax et al., 2017) and with their close contacts in those environments (Song et al., 2013; Lax et al., 2014). We recently studied a cohort of genetically unrelated individuals who lived together and identified significant numbers of shared bacteria within each household (Abeles et al., 2016). These individuals also shared viruses in both their oral and GI microbiomes (Ly et al., 2016), suggesting that passive contact amongst individuals who share the same environments may result in the sharing of viruses. Because many of the viruses inhabiting these microbiomes are bacteriophages, their ability to kill bacteria or provide them with potentially beneficial gene functions may not only shape their own microbiomes, but may also shape the microbiomes of their close contacts.

There may be no closer relationship than the bond that is formed between mother and infant. With significant focus on shared microbes amongst close contacts, the mother-infant relationship warrants further investigation because of the potential implications that shared microbes could have for infant microbiota development (Pannaraj et al., 2017), immune development (Duerkop et al., 2009; Bengmark, 2013), and for the gut-brain axis, which may affect infant neurological development (Cong et al., 2016; Sharon et al., 2016; Yang et al., 2016). Human milk harbors its own microbiome (Hunt et al., 2011), which could play a role in development of the infant GI tract microbiome via breastfeeding (Guaraldi and Salvatori, 2012; Funkhouser and Bordenstein, 2013). The existence of a microbiota in human milk began with cultivation studies demonstrating the presence of bacteria such as *Streptococcus* and *Staphylococcus* in human milk (Heikkila and Saris, 2003). These types of analyses were extended through 16S rRNA sequencing to include additional microbes such as *Bifidobacterium* and *Lactobacillus* (Martin et al., 2007; Jost et al., 2015). Finally, next-generation sequencing has extended the analysis of 16S rRNA to demonstrate the presence of substantial bacterial diversity beyond that which could be identified through prior conventional means (Hunt et al., 2011) (Backhed et al., 2015). Infants acquire nutrients such as vitamins, minerals, and fatty acids through ingestion of breast milk (Jost et al., 2015; Nongonierma and FitzGerald, 2015; Eriksen et al., 2018), and have been hypothesized to aid in the establishment of the infant GI tract microbiome (Backhed et al., 2015). Our recent studies have shown that infant GI microbiota more closely reflect the milk of their mothers than they do to unrelated mothers, indicating that microbes from human milk likely are early infant GI colonizers and may form part of the foundation of the infant GI microbiome (Pannaraj et al., 2017). This relationship is especially important because it develops through the infant ingesting human milk, which has the capacity not only to transmit bacteria, but also to potentially transmit viruses. There is some evidence that *Bifidobacteria* bacteriophages may be transmitted to infants through human milk (Duranti et al., 2017) but no specific studies characterize the human milk virome nor which of the milk virome members may be transmitted to infants. We characterized the viromes from human milk and infant stool of a cohort of mother-infant pairs, with the goal to: (1) characterize the virome of milk, (2) identify similarities and differences in the diversity of viruses in milk and infant stool viromes, (3) decipher whether milk and infant stool viromes are similar taxonomically, and (4) establish the extent to which viruses may be transmitted to the infant GI tract via milk.

## Results

### Human Subject Characteristics

We recruited 10 healthy mother-infant pairs from the community around Children’s Hospital Los Angeles and collected milk from the mothers and stool from each infant. Characteristics of mothers and infants are described in **Supplementary Table S1**. All mothers self-identified as Hispanic. Mothers’ mean age was 27 years ± standard deviation (SD) 6.8 years. Milk and stool were collected at a single time point between 4 and 10 days (mean 7.4 ± SD 1.7 days) of the infants’ birth. Infants were born between 37 and 41 weeks’ gestation. Seven of the ten infants were born via vaginal delivery, with three born via Cesarean section. Four of the mothers were exposed to peripartum antibiotics; however, none of the infants were treated with antibiotics prior to the collection of stool. Each of the mothers was actively breastfeeding at the time of the milk and stool collections, but only three of the infants had never received formula.

### Processing of Viromes and Microbiomes

We processed the viromes from milk and infant stool according to our previously described protocols through sequential filtration followed by cesium chloride density gradient centrifugation to enrich the viral fraction present in each sample type (Abeles et al., 2014; Ly et al., 2016). We produced 9,072,824 ± 749,832 (mean ± standard deviation) reads per subject and sample type and assembled those reads into larger contigs for downstream analysis. Additional details of the read numbers and the assemblies are shown in **Supplementary Table S2**. There was minimal GC content variation present amongst contigs in milk viromes, while there was substantial variability from sample to sample in median GC content of the infant stool virome contigs (**Supplementary Figure S1**). Most of the contigs had homology to viruses (85.7 ± 3.3%), some had homology to bacteria (14.3 ± 3.3%), and few had no known homologies (0.1 ± 0.1%) (**Supplementary Figure S2**). These findings together indicate that the infant stool and milk samples were substantially enriched for viruses and the GC content differences in sample types suggests greater variability in viromes in infant stool compared to milk. We also characterized the bacterial communities using the V1–V2 segment of 16S rRNA gene for comparison (Abeles et al., 2016) and produced 116,079 ± 21,005 sequence reads per subject and sample type.

### Analysis of Viral and Bacterial Alpha Diversity in Human Milk and Infant Stool

**FIGURE 1:**
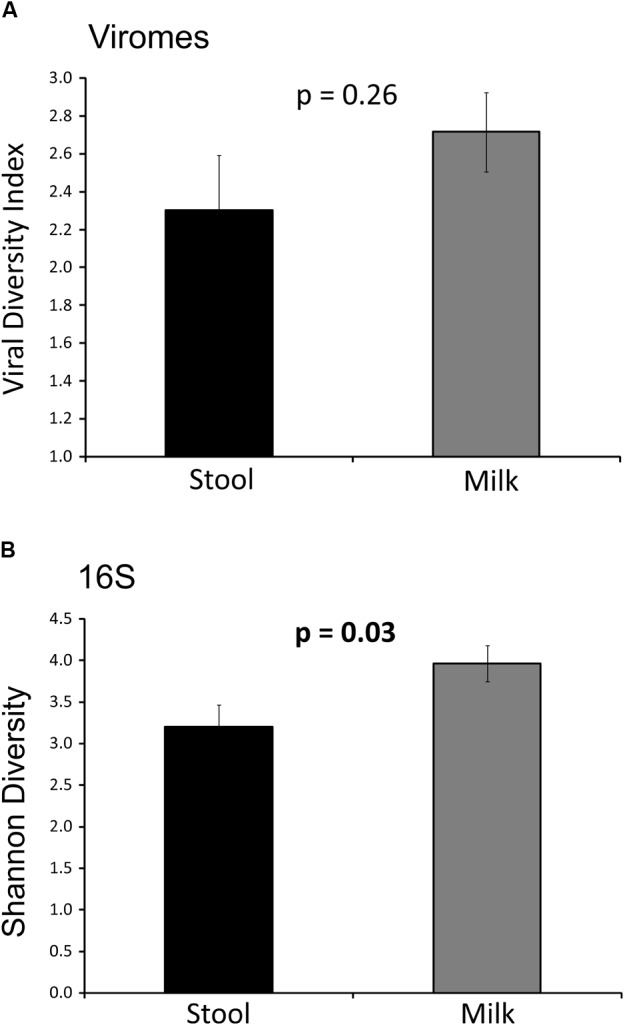
Bar graphs representing Shannon diversity and Homologous viral diversity in infant stool and human milk. **(A)** Homologous viral diversity index (± standard error) based on virome contigs. **(B)** Shannon diversity (± standard error) based on 16S rRNA, *p*-values are shown above each bar. The x-axis represents milk or infant stool, and the y-axis represents Shannon diversity or homologous viral diversity.

We characterized the alpha diversity present in the viromes and the bacterial biota of both milk and infant stool to discern whether there may be differences in the numbers of viral genotypes and bacterial operational taxonomic units (OTUs) present based on body site. Viral diversity using the homologous virus diversity index (HVDI) (Santiago-Rodriguez et al., 2015b) did not significantly differ between milk and stool (*p* = 0.26) (**Figure 1A**). We developed the HVDI based on the Shannon Index (Gotelli and Colwell, 2001) because it allows us to measure viral diversity in a similar manner by which we analyze alpha diversity in bacterial communities. In our analysis of bacterial diversity, we found that there was significantly greater diversity in maternal milk compared to infant stool (*p* = 0.03) (**Figure 1B**). While most of the milk and infant stool specimens had similar bacterial diversity amongst the subjects studied, there were a few infant stool specimens that had relatively low virome alpha diversity compared to the others studied (**Supplementary Figure S3**). There also were a slightly greater number of estimated viral genotypes present in milk than were estimated in infant stool (**Supplementary Figure S4**). These results indicate a greater level of viral diversity present in milk than in infant stool, with both below the levels of viral diversity generally observed in adult urine (Santiago-Rodriguez et al., 2015a), stool and saliva (Abeles et al., 2015).

### Comparisons of Beta Diversity in Human Milk and Infant Stool

**FIGURE 2:**
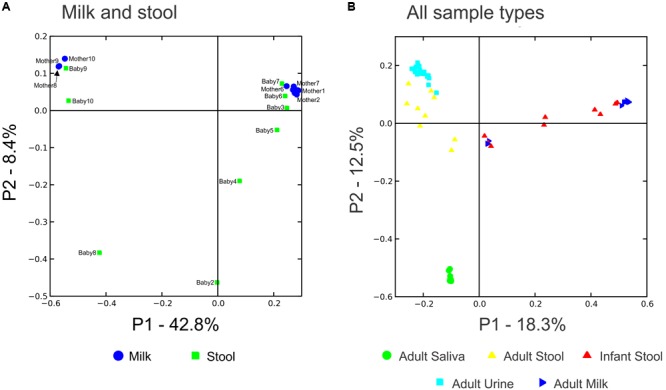
Principal coordinates analysis of beta diversity in viral communities including specimens from human milk and stool are shown in **A**, and viral communities including specimens from human milk (blue triangles), infant stool (red triangles), adult saliva (green circles), adult stool (yellow triangles), and adult urine (cyan squares) are shown in **B**.

**FIGURE 3:**
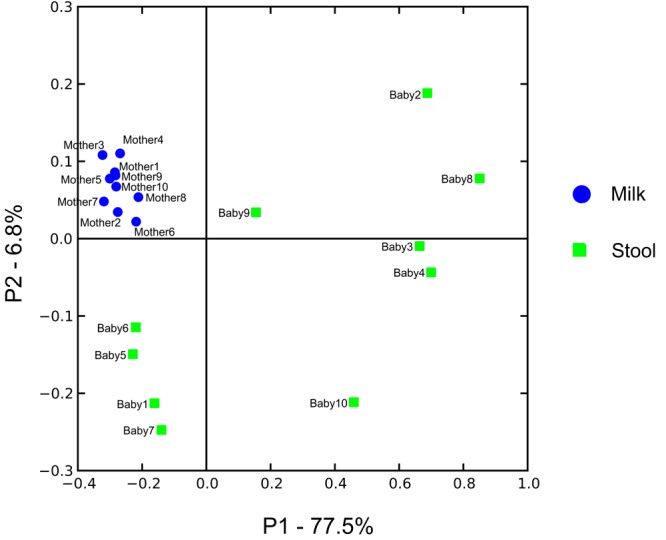
Principal coordinates analysis of beta diversity in bacterial communities (16S rRNA) of human milk (blue circles) and infant stool (green squares).

We characterized the beta diversity amongst the viromes and the bacterial biota for all mothers and infants to discern whether there were observable patterns specific to sample type and whether the samples were distinct from other sample types for which virome sequences were available. When trends in beta diversity were observed by Principal Coordinates Analysis (PCoA), the majority of the milk and infant stool viromes clustered distinctly (**Figure 2A**). Most of the milk specimens formed a homogenous cluster, but specimens from mother/infant pairs 8, 9, and 10 were somewhat distinct. The separate clustering of the specimens could not be explained by delivery method, breastfeeding exclusivity, or antibiotic exposure. These findings were consistent even when a larger group of specimens were characterized (**Figure 2B**), which demonstrated that the milk and infant stool specimens had observable differences from adult urine, saliva, and stool obtained from unrelated adults enrolled in previous studies (Robles-Sikisaka et al., 2013; Abeles et al., 2015; Santiago-Rodriguez et al., 2015a). The infant stool specimens appeared more similar to many of the milk specimens than they did to adult stool (**Figure 2B**). These results suggest that there are shared features between infant stool and milk viromes, likely as a result of milk ingestion by the infants. When examining the bacterial biota, the milk formed a rather homogenous cluster and the infant stool formed a more heterogeneous group (**Figure 3**).

### Taxonomic Analysis of Viral and Bacterial Communities

**FIGURE 4:**
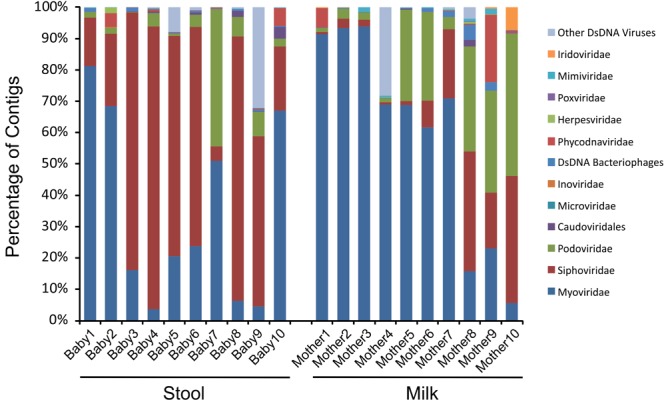
Bar graphs of putative viral family assignments. The y-axis represents the percentage of viral contigs assigned to each viral family, and the x-axis represents stool (left) from each infant studied and milk (right) from each mother studied.

**FIGURE 5:**
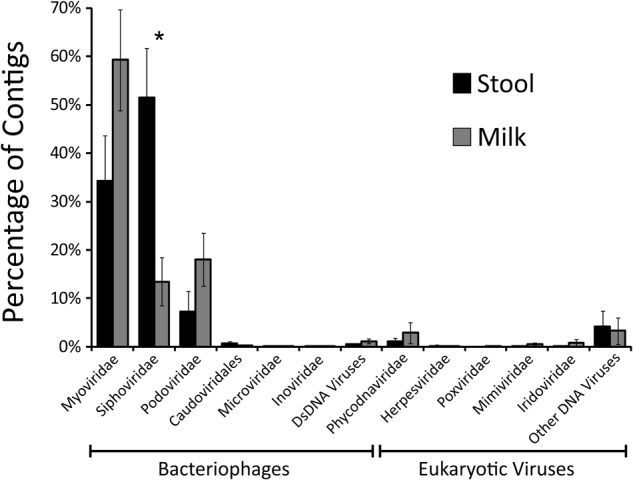
Bar graphs (± standard error) demonstrating the proportion of viral contigs assigned to specified virus families in infant stool (black bars) and human milk (gray bars). The y-axis represents the percentage of contigs assigned to each Family, and the x-axis represent the different virus families with bacteriophage families located on the left and eukaryotic virus families located on the right. The “^∗^” represents values that are statistically significant with *p*-values ≤0.01.

We next characterized the taxonomic compositions of the viromes and bacterial biota of both the infant stool and milk specimens to determine whether we may observe similar taxonomic trends amongst the mother-infant pairs. To discern trends in the taxonomic compositions of viromes, we used TBLASTX hits against the viral database present at the NCBI to identify putative viruses present in the milk and infant stool. We categorized each virus according to the family designation of their best hit (**Figure 4**). A majority of the viruses in both infant stool (95.5 ± 3.2%) and milk (95.2 ± 2.8%) were predicted to be bacteriophages, with a relatively small minority from infants (4.5 ± 3.2%) and mothers (4.8 ± 2.8%) predicted to be eukaryotic viruses (**Supplementary Figures S5**). In the infant stool, the most abundant virus family was predicted to be Siphoviridae (contractile-tailed bacteriophages that often have primarily lysogenic lifestyles) (**Figure 5**). In the milk specimens, the most abundant viruses were predicted to be from the Myoviridae family (tailed phages that often have primarily lytic lifestyles), but a few subjects also were predicted to have abundant Podoviridae (short tail stubs that often have primarily lytic lifestyles). The difference in the relative abundance of Siphoviridae between milk and infant stool was statistically significant (*p* = 0.004); however, the observed trends in differences in Myoviridae and Podoviridae between milk and stool did not meet statistical significance (*p* = 0.091 for Myoviridae and *p* = 0.130 for Podoviridae). These results highlight that there are substantial differences in the representation of bacteriophages in human milk and infant stool.

**FIGURE 6:**
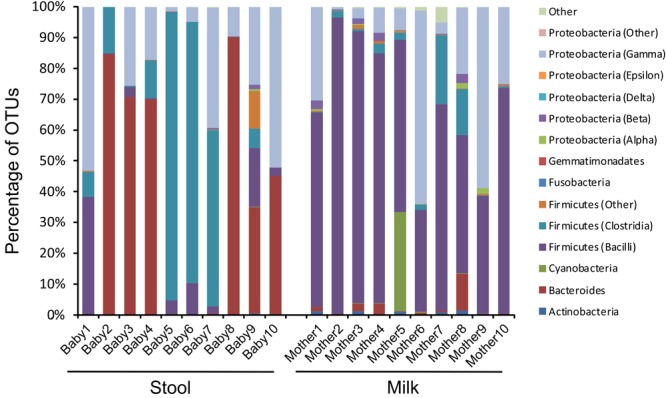
Bar graphs of bacterial communities based on 16S rRNA at the Class level. The y-axis represents the percentage of OTUs assigned to each bacteria Class, and the x-axis represents stool (left) from each infant studied and milk (right) from each mother studied. The Firmicutes from the *Bacilli* Class belong to the Genus *Streptococcus*, and the Firmicutes from the *Clostridia* Class belong to the Genus *Veillonella*.

Given the high abundance of bacteriophages among the viruses, we evaluated to see if there were similar composition changes in the bacteria biota. We observed that the infants were generally dominant in anaerobes in their stool, with most having a large abundance of *Bacteroides* and some having a large abundance of the Firmicute *Veillonella* (**Figure 6** and **Supplementary Figure S6**). In contrast, most of the milk specimens were abundant in the Firmicute *Streptococcus*. While *Streptococcus* could be observed in the stool, and both *Veillonella* and *Bacteroides* could be observed in the milk, they generally were not of high relative abundances. Mother/infant pairs 8, 9, and 10 had observable differences in virome taxonomy (**Figure 4**) and beta diversity (**Figure 2A**) compared to the other pairs, the same from mother/infant pairs also had distinctive bacterial taxonomies compared to the other pairs (**Figure 6**). These results suggest that while some bacteria may have been shared between milk and the infant GI tract, their relative abundances generally were not shared.

### Shared Viral Contigs Between Human Milk and Stool

**FIGURE 7:**
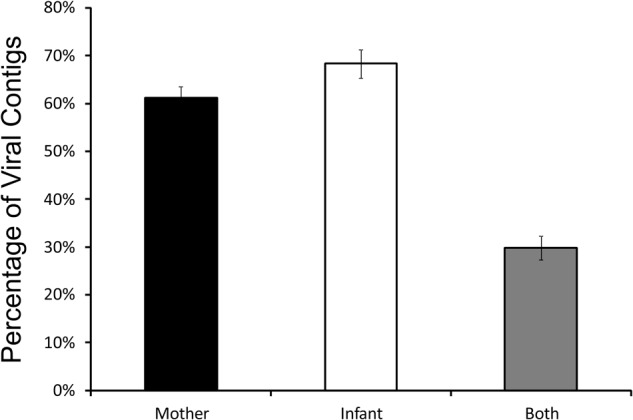
Bar graphs (± standard error) representing the relative proportion of viral contigs in each mother-infant pair assembled that include contributions from the human milk, contributions from the infant stool, or contributions from both milk and stool.

While our analysis identified distinct features of viromes in their taxonomy, alpha and beta diversity between milk and infant stool, the patterns visualized by PCoA suggested that there also were some shared features in the two specimen types. To determine whether there were shared features of these viromes between mother and infant, we quantified the numbers of viral contigs shared in each mother-infant pair. We measured this by creating global assemblies from all of the viral sequence reads obtained from both the mother and infant, and then quantified the contribution to each viral contig assembled from the mother, infant, or both the mother and infant. There were a number of contigs that were created from reads from the mother alone or the infant alone, but approximately 30% were created using reads from both the mother and infant, suggesting that these viruses were present in both (**Figure 7**). While the relative number of these contigs varied in each mother-infant pair, we identified viral contigs constructed from both milk viromes and infant stool viromes in all pairs studied (**Supplementary Figure S7**). To confirm these results, we utilized a separate method based on BLASTN homology amongst the viral contigs in both mother and infant, and performed a permutation test (Robles-Sikisaka et al., 2013) to decipher whether the proportion of highly homologous viral contigs within a mother-infant pair was significantly greater than would be observed between mother-infant pairs. In all 10 pairs, there was a significant proportion (*p* < 0.0001) of homologous viral contigs within a mother-infant pair than would be expected by comparisons between mother-infant pairs, indicating a significant conservation of viral contigs in each pair (**Table 1**). As was observed through assembly analysis (**Supplementary Figure S7**), there was substantial variation in the proportions of homologous viruses observed within each mother-infant pair (**Table 1**), suggesting that the same number of viruses were not conserved between mother and infants across the different pairs. While the proportion of homologous viruses was relatively small in some pairs (range from 3.1 to 18.4%), the results were statistically significant in each pair. We characterized some of the viral assemblies in these subjects and found that many represented bacteriophages that could be assembled with high average coverage from both the milk and the infant stool (**Supplementary Figure S8**). For example, Pair #1 shares a 15,330 nt bacteriophage in both milk and stool with significant sequence homology to *Burkholderia ambifaria* phage BcepF1 (**Supplementary Figure S8A**). Interestingly, Pair #2 shares a different 16,602 nt bacteriophage that also has significant homology to *B. ambifaria* phage BcepF1 (**Supplementary Figure S8B**). While these subjects share this likely Myovirus in both milk and infant stool, Pair #6 shares a 14,064 nt Enterobacteria phage that has high homology to Siphoviruses (**Supplementary Figure S8C**). Each of these viruses likely are of high abundance in both the milk and infant stool, as their average coverage ranges from 744X to 25,625X in each subject. We previously have demonstrated that close contacts can share portions of their viromes (Ly et al., 2016); therefore, the sharing of bacteriophages between mother and infant, is consistent with our previous findings and suggests that the sharing of viruses between close contacts is a common phenomenon.

**Table 1:** Viral homologs within and between mother-infant pairs.

By subject	Percent homologous within each pair^a^	Percent homologous between different pairs^a^	*p*-value^b^
Pair 1	18.42 ± 0.39	1.73 ± 1.34	**<0.0001**
Pair 2	15.52 ± 0.37	1.29 ± 1.04	**<0.0001**
Pair 3	13.62 ± 0.35	1.74 ± 1.41	**<0.0001**
Pair 4	4.51 ± 0.21	0.69 ± 0.58	**<0.0001**
Pair 5	12.14 ± 0.33	1.33 ± 1.01	**<0.0001**
Pair 6	14.01 ± 0.35	1.41 ± 1.07	**<0.0001**
Pair 7	9.79 ± 0.29	1.03 ± 0.86	**<0.0001**
Pair 8	3.06 ± 0.17	0.23 ± 0.45	**<0.0001**
Pair 9	5.78 ± 0.23	0.31 ± 0.60	**<0.0001**
Pair 10	5.73 ± 0.23	0.32 ± 0.46	**<0.0001**

*^*a*^Based on the mean ± standard deviation of 10,000 iterations. 1,000 random contigs were sampled per iteration. ^*b*^Empirical *p*-value based on the fraction of times the estimated percent homologous reads within each pair exceeds that for different pairs.*

## Discussion

One of the most distinct features of the human microbiome is the extent to which it may vary by body site. Indeed, it has been known for quite some time that the bacterial microbiome of the mouth is distinct from the distal colon, is distinct from the skin, and so on. This phenomenon has been investigated to a much lesser extent for the human virome. It is clear that there are distinctions between oral viromes based on their particular oral biogeographic niche (Ly et al., 2014), and that there are considerable differences in oral and stool viromes (Abeles et al., 2015), but less data exists for the viromes of other body sites. Because many of these viromes consist of considerable numbers of bacteriophages, it is reasonable to postulate that their phages may differ by body site, which is what we observed in our analysis of milk and infant stool (**Figures 4, 5**). While predicting the putative hosts of phages can be problematic, here we use their homologies to phage families to garner more information about the constituents of these phage communities. The significant contrast in the constituents of milk and infant stool strongly suggested considerable differences in these viromes, yet the proportion of shared viruses in the milk and the infant stool was higher than expected based on taxonomy. We believe that a great deal of the explanation for this discrepancy is that presence/absence of viruses in the milk and stool does not necessarily reflect relative abundances. Therefore, while the taxonomic compositions of the milk and stool remain considerably different when examining the abundances of the viruses, the presence of certain phages in both sample types demonstrates a relatedness that would be overlooked if only considering relative abundances. We speculate that this phenomenon was apparent when examining the beta diversity of the viromes, where there were substantial similarities in the milk and infant stool that was quite distinct from adult stool, salivary, and urine specimens.

We have observed significant differences in the bacterial biota on different body surfaces, but the milk and infant stool offer some of the most significant differences in virome constituents that we have observed. The relative predominance of Siphoviridae in infant stool compared to the predominance of Myoviridae in the milk was significant. While taxonomic features could represent lifestyle differences amongst the viral populations, where the viruses in milk have more predominantly lytic lifestyles compared to more lysogenic lifestyles in the infant stool, these differences also could reflect the differences in bacterial taxonomy, where *Bacteroides* and *Veillonella* were predominant in the infant stool and may have been more likely to have viruses from the Siphoviridae family. A more thorough examination of the structural features of the individual viruses identified in milk and infant stool may help to elucidate the lifestyles of these viruses by identifying whether many contain lysogenic modules. While a minority of the viruses we identified were predicted to be eukaryotic, there are methodological reasons that could lead to their underrepresentation. For example, larger viruses such as herpesviruses may be filtered out in virome preparations that use 0.2 μ filters to reduce potential bacterial contamination (Conceicao-Neto et al., 2015). Surprisingly, despite filtering viruses homologous to larger phycodnaviruses were readily identified (**Figure 4**). These viruses have previously been identified in the human oropharyngeal virome (Yolken et al., 2014), and could potentially represent contributions from infant saliva to maternal milk.

We previously examined the sharing of viruses in a cohort of unrelated cohabitating individuals and found that there was considerable sharing of viruses in relatively short time periods (Ly et al., 2016). We found evidence of sharing of both stool viruses and salivary viruses even among individuals who were not romantic couples. These data suggested that either close contact or shared environmental reservoirs resulted in the sharing of substantial numbers of viruses between individuals. That study differs in that we were measuring sharing amongst the same specimen types. For example, we measured shared stool viruses amongst the individuals in the cohort, assuming that viruses in the lower GI tract of 1 individual would have a significant propensity to colonize the lower GI tract of their housemate. In this study, we measured the sharing of viruses amongst different sample types, with the understanding that viruses that inhabit milk may transfer to the infant GI tract but not have the ability to colonize the infant distal colon. It is possible that viruses also were shared from the infant oral cavity into milk (West et al., 1979); however, because the milk volume ingested by the infant may have exceeded the spread of infant saliva into milk, transmission from milk to the infant appears more plausible. While we were able to show evidence of the same viruses colonizing the distal GI tract and the milk of the mothers, the degree of sharing was less than what we observed in the stool of unrelated housemates in that study (roughly 30% were shared at 6 months in that study (Ly et al., 2016). Because prior studies have demonstrated that bacteriophages may bind and persist on mucosal layers (Barr et al., 2013), such as those found in the distal colon, we cannot be absolutely certain that these viruses are truly replicating in the infant GI tract, but rather they may be binding and persisting on mucosal layers of the infant until those layers slough off. A longitudinal study to characterize the persistence of the phage in the infant distal colon would provide greater evidence that they may be persisting due to ongoing replication.

In our prior studies, we have sequenced viromes using Semiconductor Sequencing techniques that generally provide fewer sequence reads and less overall coverage of the viromes. In this study, we sequenced these viromes using the Illumina MiniSeq, which provided about 16–20x the coverage that we have achieved with these viromes in the past. We have a few observations from the switch in sequencing modalities, including: (1) there were not greater numbers of contigs created with the additional coverage, which supports our prior findings that the lower sequencing depth was likely adequate to characterize the contents of these viromes, (2) diversity estimates did not appear to be affected, as roughly the same proportions of reads were assembled into contigs as were found in the viromes with the lower sequencing depth, (3) the average length of the contigs was not longer with the additional sequencing depth, suggesting that sequencing depth is not the limiting factor in our ability to assemble high quality and potentially complete viral genomes, and (4) contaminants even at the relatively low levels present in these viromes become apparent with greater sequencing depth. We limited our analysis to assembled contigs >=2000 nucleotides to reduce the effects of contamination, and in doing so, identified that 85.7% of the contigs assembled had BLASTX homologs to known viral structural or replication genes.

There were a few limitations that affected the conclusions we could draw from the data in this study. Those limitations included the relatively small sample size, the cross-sectional nature of the sampling, and the characteristics of the cohort. While the intent of the study was primarily to provide the first characterization of the virome of human milk and to decipher whether viruses found in milk also could be found in the infant gut, a larger cohort may have provided greater insights into whether specific demographics and traits of the mothers and infants may have affected virome contents and virus sharing. All the mothers in this study self-identified as Hispanic, which limits whether the results of this study may be generalized across diverse populations. A longitudinal study may have definitively resolved whether viruses shared between mother and infant were the result of persistent colonization or were more likely a result of transient passage through the GI tract. Because only 4/10 infants were exclusively breastfed (**Supplementary Table S1**), while all mother/infant pairs shared viruses, it is less likely that viruses were shared solely as the result of transient GI track passage. It is possible that formula may also have a virome, which also could have contributed to the results. Despite the relatively small cohort size and variations in cohort characteristics, this study still provides the first insights into the role of the human milk virome in the establishment of the infant GI tract virome.

As scientists continue to characterize the microbiome of various body surfaces, it is becoming increasingly clear that few if any human body surfaces are not inhabited by microbes. In particular, it has been known for quite some time that human milk has both bacteria (Jeurink et al., 2013) and some viruses (Michie and Gilmour, 2001; Stiehm and Keller, 2001; Duranti et al., 2017). The presence of bacteria in milk can be traced to the third trimester of pregnancy and continues through lactation (Rodriguez, 2014). These bacteria have been hypothesized to be derived from the mother’s skin microbiota (Ramsay et al., 2004, 2005), the mother’s gut via an entero-mammary pathway (Perez et al., 2007; Jimenez et al., 2008; Abrahamsson et al., 2009; Jost et al., 2013, 2014), commensal microbiota that inhabit human breast tissue (Urbaniak et al., 2014; Xuan et al., 2014), and the infant’s oral cavity via suckling behavior (West et al., 1979). The origin of viruses in the infant GI tract has been hypothesized to occur through vertical transmission from mother to infant, with breast milk as the likely means of the transmission (Duranti et al., 2017). The data presented in this study supports that breast milk may be responsible for a portion of the viruses identified in the infant GI tract.

The relationship between mother and infant has gained greater attention as researchers focus on the role of the microbiome in gut-brain axis development and the role of the microbiome in the development of the infant immune system (Duerkop et al., 2009; Bengmark, 2013; Cong et al., 2016; Yang et al., 2016). Prior studies show that infants born vaginally are colonized by a number of microbes that ultimately may play a role in infant development (Bokulich et al., 2016). The data presented here add an additional layer of complexity to the potential role of the microbiome in infant development, as it has identified a virome unique to milk that may then be transmitted to the infant. While most of the viruses identified in this study were bacteriophages, there were some Eukaryotic viruses also identified, and these have previously been shown to play some role in the development of the immune system in germ-free mice (murine norovirus). Even if most of the viruses observed in this study are not directly involved in immune development or communication along the gut-brain axis, their role as natural perturbations could be important in determining the bacterial constituents of the infant GI tract in its earliest stage of development.

## Materials and Methods

### Human Subjects and Culture Conditions

Human subject involvement in this study was approved by the Institutional Review Board at Children’s Hospital Los Angeles. Written informed consent was obtained from all mothers and financial compensation was provided for their participation in this study. We recruited healthy mothers and their full-term infants from the Los Angeles community and recorded mother and infant ages, comorbidities, current and recent antibiotic use, and infant feeding characteristics. We collected milk from mothers and stool from the infants’ soiled diapers during study visits. Visits occurred during all times of the day depending on the mother’s availability. Mothers were instructed to clean the breast per their normal routine prior to feeding. Milk (1–2 ounces) was obtained either through manual expression or electric breast pumps using sterilized connectors and sterile bottles for collection. We collected from a total of 10 mother/infant pairs, and both milk and stool was stored at -80°C until processed in this study.

### Analysis of 16S rRNA

Genomic DNA was prepared from the maternal milk using the Qiagen QIAamp DNA MINI kit (Qiagen, Valencia, CA, United States) and from the stool using the QIAamp DNA Stool MINI kit. Each sample was subjected to a bead beating step prior to nucleic acid extraction using Lysing Matrix-B (MP Bio, Santa Ana, CA, United States). We amplified the bacterial 16S rRNA gene V1-V2 hypervariable region using the forward primer 8F (AGAGTTTGATCCTGGCTCAG) fused with the Ion Torrent Adaptor A sequence and one of 70 unique 10 base pair barcodes, and reverse primer 357R (CTGCTGCCTYCCGTA) fused with the Ion Torrent Adaptor P1 from each subject and sample type (Whiteley et al., 2012). PCR reactions were performed using Platinum High Fidelity PCR SuperMix (Invitrogen) with the following cycling parameters: 94°C for 10 min, followed by 30 cycles of 94°C for 30 s, 53°C for 30 s, 72°C for 30 s, and a final elongation step of 72°C for 10 min. Resulting amplicons were purified on a 2% agarose gel stained with SYBR Safe (Invitrogen) using the Qiagen MinElute PCR Purification Kit. Amplicons were further purified with Ampure XP beads (Beckman-Coulter), and molar equivalents were determined for each sample by quantifying the amplicons using PicoGreen (Invitrogen) using a plate reader. Samples were pooled into equal molar proportions and sequenced on 316 chips using an Ion Torrent PGM according to manufacturer’s instructions (Life Technologies) (Rothberg et al., 2011). Resulting sequence reads were removed from the analysis if they were <180 nucleotides or >400 nucleotides, had any barcode or primer errors, contained any ambiguous characters, or contained any stretch of >8 consecutive homopolymers. Sequences then were trimmed according to any site that had a Phred Score of less than 15. Sequences then were assigned to their respective samples based on a 10 nucleotide barcode sequence using Ion Assist^1^.

We sequenced an average of 116,079 reads from each sample, and analyzed the sequence data using Quantitative Insights Into Microbial Ecology (QIIME 1.5) (Caporaso et al., 2010). Representative OTUs from each set were chosen at a minimum sequence identity of 97% using the QIIME script pick_otus_through_otu_table, which uses the Greengenes database (DeSantis et al., 2006). PCoA was performed based on beta diversity using weighted UniFrac distances (Lozupone et al., 2006) using the QIIME script beta_diversity_through_plots. The results of the beta diversity distance matrices were used to determine the weighted UniFrac distances between different samples and sample groups. Alpha diversity based on the Shannon Index (Gotelli and Colwell, 2001) also was performed using the QIIME pipeline. Differences in the relative abundances of taxa between subject groups were determined using the Mann Whitney U test using MaxStat Pro^2^.

### Virome Preparation and Sequencing

Stool viromes were prepared by diluting 0.4 g of stool in 4 mL of SM buffer (100 mM NaCl, 8 mM MgSO4, and 50 mM Tris pH 7.5), vortexing for 40 min to separate viral particles, spun at 4,000 × *g* for 10 min at 4°C to pellet the remaining solid material, and the supernatant saved from each specimen. The milk specimens were centrifuged at 4,000 × *g* for 10 min at 4°C, and the supernatant was then treated in an identical manner as we have developed for saliva specimens (Abeles et al., 2014). Supernatants from stool and milk were filtered sequentially using 0.45 and 0.2 μm cellulose acetate filters (GE Healthcare Life Sciences) to remove cellular and other debris, and then purified on a cesium chloride gradient according to previously described protocols (Pride et al., 2012). Only the fraction with a density corresponding to most known bacteriophages (Murphy et al., 1995) was retained, further purified on Amicon YM-100 protein purification columns (Millipore, Inc.), treated with 2 units of DNase I, and subjected to lysis and DNA purification using the Qiagen UltraSens Virus kit (Qiagen). Recovered DNA was screened for the presence of contaminating bacterial nucleic acids by quantitative 16S rRNA gene PCR using primers 8F (AGAGTTTGATCCTGGCTCAG) and 357R (CTGCTGCCTYCCGTA) in Power SYBR Green PCR Mastermix (Thermo Fisher Scientific) (Abeles et al., 2015). No products were detected in any of the viromes after 30 cycles, which does not exclude the presence of contaminating bacterial nucleic acids, but indicates that they were not present at dominant levels. Viral DNA then was amplified using GenomiPhi Hy MDA amplification (GE Healthcare Life Sciences), and specimens prepared for sequencing using the Nextera DNA Library Prep XT kit (Illumina, Inc) according to manufacturer instructions. The size of products were determined using a High Sensitivity DNA Kit on a Bioanalyzer (Agilent Technologies), and quantified using a High Sensitivity Double Stranded DNA kit on a Qubit Fluorometer (Thermo Fisher Scientific). DNA from each specimen was pooled into equimolar proportions and sequenced on the Illumina MiniSeq Instrument (Illumina, Inc), producing an average of 9,072,824 paired-end reads per specimen. For quality control on the sequence reads, we performed the following steps: (1) we trimmed sequence reads with Phred scores <30, (2) we removed any reads with ≥8 consecutive homopolymers, (3) we removed reads <50 nucleotides or >300 nucleotides in length, and (4) we removed reads that contained any ambiguous nucleotides. Each virome was screened for contaminating nucleic acids using BLASTN analysis (*E*-score <10^-5^) against the human reference database available at ftp://ftp.ncbi.nlm.nih.gov/genomes/H_sapiens/. Any reads with significant sequence similarities to human sequences were removed prior to further analysis using Ion Assist^3^.

### Virome Analysis

All reads were assembled using CLC Genomics Workbench 9.5.3 (Qiagen) based on 98% identity with a minimum of 50% read overlap, which were more stringent than criteria developed to discriminate between highly related viruses (Breitbart et al., 2002). Because the average and median read lengths were 150 nucleotides, the minimum tolerable overlap was approximately 75 nucleotides. Assemblies created containing reads from milk and infant stool were assembled using the same parameters. The consensus sequence for each contig was constructed according to majority rule, and any contigs <2000 nucleotides were removed prior to further analysis. Contigs were annotated using BLASTX against the NCBI NR database with an *E*-value cutoff value of 10^-5^. Specific viral sequences were identified using Ion Assist^3^ by parsing BLASTX results for known viral genes including replication, structural, transposition, restriction/modification, hypothetical, and other genes previously found in viruses for which the *E*-value was at least 10^-5^. Each individual virome contig was annotated using this technique; however, if the best hit for any portion of the contig was to a gene with no known function, lower level hits were used as long as they had known function and still met the *E*-value cutoff. ORF prediction was performed using FGenesV (Softberry Inc, Mount Kisco, NY, United States), and putative functions assigned by BLASTP homology against the NR database (*E*-score <10^-5^). If the best hit was to a gene with no known function, lower level hits were used for the annotation as long as they had known putative function and still met the *E*-score cutoff (10^-5^). Virus types were determined by parsing the virus families from the TBLASTX best hits of each viral contigs with an *E*-value <10^-20^. Statistically significant differences in the representation of viruses between groups were determined by two-tailed *t*-tests.

Analysis of shared sequence similarities present in each virome was performed by creating custom BLAST databases for each virome, comparing each database with all other viromes using BLASTN analysis (*E*-value <10^-10^), and these compiled data used to calculate beta diversity with Bray Curtis distances using QIIME (Caporaso et al., 2010). These distances were used as input for PCoA. We determined putative sharing of viruses by constructing assemblies from both the milk and infant stool reads from each subject, and then deciphered the contribution to each resulting contig from milk reads and the infant stool reads, similar to techniques we have previously described (Abeles et al., 2014; Ly et al., 2016). We utilized this technique to decipher those contigs that were unique to milk or infant stool, and those shared between milk and infant stool in each mother-infant pair. Statistical significance was determined by comparisons between groups by the Mann Whitney *U* test using MaxStat Pro^4^. We also performed a permutation test (10,000 iterations) to assess whether infant stool and milk viromes had significant overlap in each mother-infant pair using Ion Assist^4^ (Robles-Sikisaka et al., 2013, 2014; Abeles et al., 2014; Ly et al., 2014; Naidu et al., 2014). We simulated the distribution of the fraction of shared virome homologs between milk and infant stool within each mother-infant pair. For each pair, we computed the summed fraction of shared homologs using 1000 random contigs between randomly chosen between milk and infant stool of different mother-infant pairs, and from these computed an empirical null distribution of our statistic of interest (the fraction of shared homologs). The simulated statistics within each pair across all time points were referred to the null distribution of inter-pair comparisons, and the *p*-value was computed as the fraction of times the simulated statistic for the each exceeded the observed statistic.

### Viral Diversity

To measure alpha diversity in the viral communities, we utilized a technique termed the HVDI. The technique is based on finding high levels of homology amongst contigs within viromes that likely belong to the same virus but were placed into separate contigs due to the limitations of the assembly process (Abeles et al., 2014). Virome reads were assembled using 98% identify over a minimum of 50% of the read length using CLC Genomics Workbench 4.65 (CLC bio USA, Cambridge, MA, United States), and the resulting contig spectra utilized as the primary input for the index. We created custom nucleotide BLAST databases for each subject that contained all their contigs. We then used BLASTN analysis to find high levels of homology (e-score <10^-20^) between different contigs within the same subject. We accepted only high levels of homology that spanned at least 50% of the length of the shorter contig being compared. All contigs in each subject were treated as nodes and those contigs that had high homology to other contigs in the same subject were added to a network by directing edges between the nodes. After evaluating homologies among all intra-subject contigs, networks formed from directed edges/nodes were assigned to individual viruses and nodes with no associations were considered singular viruses. For each resulting network, we added the number of reads assigned to each node on the network and the combined number of reads was used to represent the relative abundance of the virus represented by that network. The relative abundances of all viruses were calculated using this technique, and a new contig spectra representing the viral population in each subject was formed. The contig spectra from each subject then were used as surrogates for population structures and input directly into the Shannon Index (Gotelli and Colwell, 2001) to estimate diversity.

## Declarations

### Ethics Approval and Consent to Participate

Subject recruitment and enrollment were approved by the Human Research Protection Programs at the Children’s Hospital Los Angeles under project # CCI-12-00044. All subjects signed written informed consent indicating their willingness to participate in this study and willingness to allow us to publish our findings. Each subject was compensated for their participation in this study.

### Consent to Publish

The consent forms signed by each participant included their consent to allow us to publish our findings.

## Availability of Data and Materials

All sequences are available for download in the Sequence Read Archive under accession number SRP127558 (https://www.ncbi.nlm.nih.gov/sra/?term=SRP127558). Ion Assist can be found at www.thepridelaboratory.org and runs on Windows XP or higher.

## Author Contributions

PP and DP conceived and designed the experiments. PP and CC recruited subjects. ML, MS, KJ, and AS performed the experiments. PP and DP analyzed the data. PP contributed reagents, performed the examinations. PP, GA, and DP wrote and critically reviewed the manuscript.

## Conflict of Interest Statement

PP receives funding from AstraZeneca and MedImmune for clinical research studies. The other authors declare that the research was conducted in the absence of any commercial or financial relationships that could be construed as a potential conflict of interest.

## Footnotes

1 www.thepridelaboratory.org

2 www.maxstat.de

3 www.thepridelaboratory.org

4 www.maxstat.de

## Supplementary Material

FIGURE S1 Box and whiskers plots demonstrating the percent G + C content amongst the virome reads recovered from infant stool (left) and human milk (right). The y-axis represents the percent G + C content.

FIGURE S2 Bar graphs representing the proportion of contigs from infant stool and human milk with BLASTX hits to known viruses, bacteria, or without BLASTX hits. The y-axis represents the percentage of total contigs and the x-axis represents each infant stool (left) and milk (right) specimen.

FIGURE S3 Bar graphs representing Shannon diversity and Homologous viral diversity in infant stool and human milk. **(A)** Homologous viral diversity index based on virome contigs for all infants and mothers, **(B)** Shannon diversity based on 16S rRNA for all infants and mothers. The x-axis represents milk (right) or infant stool (left), and the y-axis represents Shannon diversity or homologous viral diversity.

FIGURE S4 Bar graphs (± standard error) representing the estimated number of virus genotypes identified in infant stool and human milk. The x-axis represents milk or infant stool, and the y-axis represents estimated virus genotypes determined by the homologous virus diversity index. The *p*-value is shown above.

FIGURE S5 Bar graphs (± standard error) representing the proportion of contigs from infant stool and human milk with TBLASTX homology to bacteriophage and eukaryotic virus families. The percentage of contigs is represented on the y-axis, and the infant stool and milk specimens are shown on the x-axis. *p*-values ≤0.001 are represented by “^∗∗^”.

FIGURE S6 Bar graphs (± standard error) demonstrating the proportion of OTUs assigned to specific bacteria Classes in infant stool (black bars) and human milk (gray bars). The y-axis represents the percentage of OTUs assigned to each Class, and the x-axis represent the different bacteria Classes. The “^∗^” represents values that are statistically significant with *p*-values ≤0.01, the “^∗∗^” represents *p*-values ≤0.001, and the “^∗∗∗^” represents *p*-values ≤0.0001. The Firmicutes from the *Bacilli* Class belong to the Genus *Streptococcus*, and the Firmicutes from the *Clostridia* Class belong to the Genus *Veillonella*.

FIGURE S7 Bar graphs representing the relative proportion of viral contigs in each mother-infant pair assembled that include contributions from the human milk, contributions from the infant stool, or contributions from both milk and stool. The y-axis represents the percentage of viral contigs assembled, and the x-axis represents the contribution to viral contigs from the mother (black), infant (white), or both (gray).

FIGURE S8 Assemblies of viral contigs from select mother-infant pairs. **(A)** represents a 15,330 nt phage from mother-infant Pair #1, **(B)** represents a 16,602 phage from mother-infant Pair #2, and **(C)** represents a 14,064 phage from mother-infant Pair #6. The average coverage from the infant stool and the human milk is shown on the right of each panel. The portions of the contig identified in the milk and stool are represented by the colored boxes. Putative ORFs and their directions are represented by the arrows and their annotations are represented in each panel. The length of the contig is denoted at the top.

TABLE S1 Characteristics of study participants at time of specimen collection.

TABLE S2 Contig metrics from milk and stool.

## Abbreviations

PCoA: principal coordinates analysis

## References

[B1] Abeles S. R. Jones M. B. Santiago-Rodriguez T. M. Ly M. Klitgord N. Yooseph S. (2016). Microbial diversity in individuals and their household contacts following typical antibiotic courses. *Microbiome* 4:39. 10.1186/s40168-016-0187-9 27473422 PMC4967329

[B2] Abeles S. R. Ly M. Santiago-Rodriguez T. M. Pride D. T. (2015). Effects of long term antibiotic therapy on human oral and fecal viromes. *PLoS One* 10:e0134941. 10.1371/journal.pone.0134941 26309137 PMC4550281

[B3] Abeles S. R. Robles-Sikisaka R. Ly M. Lum A. G. Salzman J. Boehm T. K. (2014). Human oral viruses are personal, persistent and gender-consistent. *ISME J.* 8 1753–1767. 10.1038/ismej.2014.31 24646696 PMC4139723

[B4] Abrahamsson T. R. Sinkiewicz G. Jakobsson T. Fredrikson M. Bjorksten B. (2009). Probiotic lactobacilli in breast milk and infant stool in relation to oral intake during the first year of life. *J. Pediatr. Gastroenterol. Nutr.* 49 349–354. 10.1097/MPG.0b013e31818f091b 19525871

[B5] Backhed F. Roswall J. Peng Y. Feng Q. Jia H. Kovatcheva-Datchary P. (2015). Dynamics and stabilization of the human gut microbiome during the first year of life. *Cell Host Microbe* 17 690–703. 10.1016/j.chom.2015.04.004 25974306

[B6] Barr J. J. Auro R. Furlan M. Whiteson K. L. Erb M. L. Pogliano J. (2013). Bacteriophage adhering to mucus provide a non-host-derived immunity. *Proc. Natl. Acad. Sci. U.S.A.* 110 10771–10776. 10.1073/pnas.1305923110 23690590 PMC3696810

[B7] Bengmark S. (2013). Gut microbiota, immune development and function. *Pharmacol. Res.* 69 87–113. 10.1016/j.phrs.2012.09.002 22989504

[B8] Bokulich N. A. Chung J. Battaglia T. Henderson N. Jay M. Li H. (2016). Antibiotics, birth mode, and diet shape microbiome maturation during early life. *Sci. Transl. Med.* 8:343ra82. 10.1126/scitranslmed.aad7121 27306664 PMC5308924

[B9] Breitbart M. Haynes M. Kelley S. Angly F. Edwards R. A. Felts B. (2008). Viral diversity and dynamics in an infant gut. *Res. Microbiol* 159 367–373. 10.1016/j.resmic.2008.04.006 18541415

[B10] Breitbart M. Salamon P. Andresen B. Mahaffy J. M. Segall A. M. Mead D. (2002). Genomic analysis of uncultured marine viral communities. *Proc. Natl. Acad. Sci. U.S.A.* 99 14250–14255. 10.1073/pnas.202488399 12384570 PMC137870

[B11] Caporaso J. G. Kuczynski J. Stombaugh J. Bittinger K. Bushman F. D. Costello E. K. (2010). QIIME allows analysis of high-throughput community sequencing data. *Nat. Meth.* 7 335–336. 10.1038/nmeth.f.303 20383131 PMC3156573

[B12] Columpsi P. Sacchi P. Zuccaro V. Cima S. Sarda C. Mariani M. (2016). Beyond the gut bacterial microbiota: The gut virome. *J. Med. Virol.* 88 1467–1472. 10.1002/jmv.24508 26919534 PMC7166815

[B13] Conceicao-Neto N. Zeller M. Lefrere H. De Bruyn P. Beller L. Deboutte W. (2015). Modular approach to customise sample preparation procedures for viral metagenomics: a reproducible protocol for virome analysis. *Sci. Rep.* 5 16532. 10.1038/srep16532 26559140 PMC4642273

[B14] Cong X. Xu W. Romisher R. Poveda S. Forte S. Starkweather A. (2016). Gut microbiome and infant health: brain-gut-microbiota axis and host genetic factors. *Yale J. Biol. Med.* 89 299–308. 27698614 PMC5045139

[B15] DeSantis T. Z. Hugenholtz P. Larsen N. Rojas M. Brodie E. L. Keller K. (2006). Greengenes, a chimera-checked 16S rRNA gene database and workbench compatible with ARB. *Appl. Environ. Microbiol.* 72 5069–5072. 10.1128/AEM.03006-05 16820507 PMC1489311

[B16] Ding T. Schloss P. D. (2014). Dynamics and associations of microbial community types across the human body. *Nature* 509 357–360. 10.1038/nature13178 24739969 PMC4139711

[B17] Duerkop B. A. Vaishnava S. Hooper L. V. (2009). Immune responses to the microbiota at the intestinal mucosal surface. *Immunity* 31 368–376. 10.1016/j.immuni.2009.08.009 19766080

[B18] Duranti S. Lugli G. A. Mancabelli L. Armanini F. Turroni F. James K. (2017). Maternal inheritance of bifidobacterial communities and bifidophages in infants through vertical transmission. *Microbiome* 5:66. 10.1186/s40168-017-0282-6 28651630 PMC5485682

[B19] Edelblum K. L. Sharon G. Singh G. Odenwald M. A. Sailer A. Cao S. (2017). The microbiome activates CD4 T-cell-mediated immunity to compensate for increased intestinal permeability. *Cell. Mol. Gastroenterol. Hepatol.* 4 285–297. 10.1016/j.jcmgh.2017.06.001 28795125 PMC5540699

[B20] Eloe-Fadrosh E. A. Rasko D. A. (2013). The human microbiome: from symbiosis to pathogenesis. *Annu. Rev. Med.* 64 145–163. 10.1146/annurev-med-010312-133513 23327521 PMC3731629

[B21] Eriksen K. G. Christensen S. H. Lind M. V. Michaelsen K. F. (2018). Human milk composition and infant growth. *Curr. Opin. Clin. Nutr. Metab. Care* 21 200–206. 10.1097/MCO.0000000000000466 29461264

[B22] Funkhouser L. J. Bordenstein S. R. (2013). Mom knows best: the universality of maternal microbial transmission. *PLoS Biol.* 11:e1001631. 10.1371/journal.pbio.1001631 23976878 PMC3747981

[B23] Gensollen T. Iyer S. S. Kasper D. L. Blumberg R. S. (2016). How colonization by microbiota in early life shapes the immune system. *Science* 352 539–544. 10.1126/science.aad9378 27126036 PMC5050524

[B24] Gotelli N. J. Colwell R. K. (2001). Quantifying biodiversity: procedures and pitfalls in the measurement and comparison of species richness. *Ecol. Lett.* 4 379–391. 10.1046/j.1461-0248.2001.00230.x

[B25] Guaraldi F. Salvatori G. (2012). Effect of breast and formula feeding on gut microbiota shaping in newborns. *Front. Cell. Infect. Microbiol.* 2:94. 10.3389/fcimb.2012.00094 23087909 PMC3472256

[B26] Hannigan G. D. Meisel J. S. Tyldsley A. S. Zheng Q. Hodkinson B. P. Sanmiguel A. J. (2015). The human skin double-stranded DNA virome: topographical and temporal diversity, genetic enrichment, and dynamic associations with the host microbiome. *MBio* 6:e1578-15. 10.1128/mBio.01578-15 26489866 PMC4620475

[B27] Heikkila M. P. Saris P. E. (2003). Inhibition of *Staphylococcus aureus* by the commensal bacteria of human milk. *J. Appl. Microbiol.* 95 471–478. 10.1046/j.1365-2672.2003.02002.x 12911694

[B28] Hunt K. M. Foster J. A. Forney L. J. Schutte U. M. Beck D. L. Abdo Z. (2011). Characterization of the diversity and temporal stability of bacterial communities in human milk. *PLoS One* 6:e21313. 10.1371/journal.pone.0021313 21695057 PMC3117882

[B29] Jeurink P. V. van Bergenhenegouwen J. Jimenez E. Knippels L. M. Fernandez L. Garssen J. (2013). Human milk: a source of more life than we imagine. *Benef. Microbes* 4 17–30. 10.3920/BM2012.0040 23271066

[B30] Jimenez E. Fernandez L. Maldonado A. Martin R. Olivares M. Xaus J. (2008). Oral administration of *Lactobacillus* strains isolated from breast milk as an alternative for the treatment of infectious mastitis during lactation. *Appl. Environ. Microbiol.* 74 4650–4655. 10.1128/AEM.02599-07 18539795 PMC2519365

[B31] Jost T. Lacroix C. Braegger C. Chassard C. (2013). Assessment of bacterial diversity in breast milk using culture-dependent and culture-independent approaches. *Br. J. Nutr.* 110 1253–1262. 10.1017/S0007114513000597 23507238

[B32] Jost T. Lacroix C. Braegger C. Chassard C. (2015). Impact of human milk bacteria and oligosaccharides on neonatal gut microbiota establishment and gut health. *Nutr. Rev.* 73 426–437. 10.1093/nutrit/nuu016 26081453

[B33] Jost T. Lacroix C. Braegger C. P. Rochat F. Chassard C. (2014). Vertical mother-neonate transfer of maternal gut bacteria via breastfeeding. *Environ. Microbiol.* 16 2891–2904. 10.1111/1462-2920.12238 24033881

[B34] Laforest-Lapointe I. Arrieta M. C. (2017). Patterns of early-life gut microbial colonization during human immune development: an ecological perspective. *Front. Immunol.* 8:788. 10.3389/fimmu.2017.00788 28740492 PMC5502328

[B35] Lax S. Sangwan N. Smith D. Larsen P. Handley K. M. Richardson M. (2017). Bacterial colonization and succession in a newly opened hospital. *Sci. Transl. Med.* 9:eaah6500. 10.1126/scitranslmed.aah6500 28539477 PMC5706123

[B36] Lax S. Smith D. P. Hampton-Marcell J. Owens S. M. Handley K. M. Scott N. M. (2014). Longitudinal analysis of microbial interaction between humans and the indoor environment. *Science* 345 1048–1052. 10.1126/science.1254529 25170151 PMC4337996

[B37] Lim E. S. Zhou Y. Zhao G. Bauer I. K. Droit L. Ndao I. M. (2015). Early life dynamics of the human gut virome and bacterial microbiome in infants. *Nat. Med.* 21 1228–1234. 10.1038/nm.3950 26366711 PMC4710368

[B38] Lozupone C. Hamady M. Knight R. (2006). UniFrac–an online tool for comparing microbial community diversity in a phylogenetic context. *BMC Bioinformatics* 7:371. 10.1186/1471-2105-7-371 16893466 PMC1564154

[B39] Ly M. Abeles S. R. Boehm T. K. Robles-Sikisaka R. Naidu M. Santiago-Rodriguez T. (2014). Altered oral viral ecology in association with periodontal disease. *MBio* 5:e1133-14. 10.1128/mBio.01133-14 24846382 PMC4030452

[B40] Ly M. Jones M. B. Abeles S. R. Santiago-Rodriguez T. M. Gao J. Chan I. C. (2016). Transmission of viruses via our microbiomes. *Microbiome* 4:64. 10.1186/s40168-016-0212-z 27912785 PMC5134127

[B41] Martin R. Heilig H. G. Zoetendal E. G. Jimenez E. Fernandez L. Smidt H. (2007). Cultivation-independent assessment of the bacterial diversity of breast milk among healthy women. *Res. Microbiol.* 158 31–37. 10.1016/j.resmic.2006.11.004 17224259

[B42] Michie C. A. Gilmour J. (2001). Breast feeding and the risks of viral transmission. *Arch. Dis. Child.* 84 381–382. 10.1136/adc.84.5.381 11316675 PMC1718779

[B43] Murphy F. A. Fauquet C. M. Bishop D. H. L. Ghabrial S. A. Jarvis A. W. Martelli G. P. (1995). *Virus Taxonomy: Sizth Report of the International Committee on Taxonomy of Viruses* Vol. 10 New York, NY: Springer-Verlag 10.1007/978-3-7091-6607-9

[B44] Naidu M. Robles-Sikisaka R. Abeles S. R. Boehm T. K. Pride D. T. (2014). Characterization of bacteriophage communities and CRISPR profiles from dental plaque. *BMC Microbiol.* 14:175. 10.1186/1471-2180-14-175 24981669 PMC4104742

[B45] Nongonierma A. B. FitzGerald R. J. (2015). Bioactive properties of milk proteins in humans: a review. *Peptides* 73 20–34. 10.1016/j.peptides.2015.08.009 26297879

[B46] Pannaraj P. S. Li F. Cerini C. Bender J. M. Yang S. Rollie A. (2017). Association between breast milk bacterial communities and establishment and development of the infant gut microbiome. *JAMA Pediatr.* 171 647–654. 10.1001/jamapediatrics.2017.0378 28492938 PMC5710346

[B47] Perez P. F. Dore J. Leclerc M. Levenez F. Benyacoub J. Serrant P. (2007). Bacterial imprinting of the neonatal immune system: lessons from maternal cells? *Pediatrics* 119 e724-32. 17332189 10.1542/peds.2006-1649

[B48] Pride D. T. Salzman J. Haynes M. Rohwer F. Davis-Long C. White R. A. (2012). Evidence of a robust resident bacteriophage population revealed through analysis of the human salivary virome. *ISME J.* 6 915–926. 10.1038/ismej.2011.169 22158393 PMC3329113

[B49] Ramsay D. T. Kent J. C. Owens R. A. Hartmann P. E. (2004). Ultrasound imaging of milk ejection in the breast of lactating women. *Pediatrics* 113 361–367. 10.1542/peds.113.2.361 14754950

[B50] Ramsay D. T. Mitoulas L. R. Kent J. C. Larsson M. Hartmann P. E. (2005). The use of ultrasound to characterize milk ejection in women using an electric breast pump. *J. Hum. Lact.* 21 421–428. 10.1177/0890334405280878 16280558

[B51] Reyes A. Blanton L. V. Cao S. Zhao G. Manary M. Trehan I. (2015). Gut DNA viromes of Malawian twins discordant for severe acute malnutrition. *Proc. Natl. Acad. Sci. U.S.A.* 112 11941–11946. 10.1073/pnas.1514285112 26351661 PMC4586842

[B52] Robles-Sikisaka R. Ly M. Boehm T. Naidu M. Salzman J. Pride D. T. (2013). Association between living environment and human oral viral ecology. *ISME J.* 7 1710–1724. 10.1038/ismej.2013.63 23598790 PMC3749502

[B53] Robles-Sikisaka R. Naidu M. Ly M. Salzman J. Abeles S. R. Boehm T. K. (2014). Conservation of streptococcal CRISPRs on human skin and saliva. *BMC Microbiol.* 14:146. 10.1186/1471-2180-14-146 24903519 PMC4063239

[B54] Rodriguez J. M. (2014). The origin of human milk bacteria: is there a bacterial entero-mammary pathway during late pregnancy and lactation? *Adv. Nutr.* 5 779–784. 10.3945/an.114.007229 25398740 PMC4224214

[B55] Rothberg J. M. Hinz W. Rearick T. M. Schultz J. Mileski W. Davey M. (2011). An integrated semiconductor device enabling non-optical genome sequencing. *Nature* 475 348–352. 10.1038/nature10242 21776081

[B56] Santiago-Rodriguez T. M. Ly M. Bonilla N. Pride D. T. (2015a). The human urine virome in association with urinary tract infections. *Front. Microbiol.* 6:14. 10.3389/fmicb.2015.00014 25667584 PMC4304238

[B57] Santiago-Rodriguez T. M. Ly M. Daigneault M. C. Brown I. H. Mcdonald J. A. Bonilla N. (2015b). Chemostat culture systems support diverse bacteriophage communities from human feces. *Microbiome* 3:58. 10.1186/s40168-015-0124-3 26549756 PMC4638026

[B58] Sanz Y. Olivares M. Moya-Perez A. Agostoni C. (2015). Understanding the role of gut microbiome in metabolic disease risk. *Pediatr. Res.* 77 236–244. 10.1038/pr.2014.170 25314581

[B59] Sauvage V. Laperche S. Cheval J. Muth E. Dubois M. Boizeau L. (2016). Viral metagenomics applied to blood donors and recipients at high risk for blood-borne infections. *Blood Transfus.* 14 400–407. 10.2450/2016.0160-15 27136432 PMC5016298

[B60] Sharon G. Sampson T. R. Geschwind D. H. Mazmanian S. K. (2016). The central nervous system and the gut microbiome. *Cell* 167 915–932. 10.1016/j.cell.2016.10.027 27814521 PMC5127403

[B61] Song S. J. Lauber C. Costello E. K. Lozupone C. A. Humphrey G. Berg-Lyons D. (2013). Cohabiting family members share microbiota with one another and with their dogs. *Elife* 2:e00458. 10.7554/eLife.00458 23599893 PMC3628085

[B62] Stiehm E. R. Keller M. A. (2001). Breast milk transmission of viral disease. *Adv. Nutr. Res.* 10 105–122. 11795036 10.1007/978-1-4615-0661-4_5

[B63] Urbaniak C. Cummins J. Brackstone M. Macklaim J. M. Gloor G. B. Baban C. K. (2014). Microbiota of human breast tissue. *Appl. Environ. Microbiol.* 80 3007–3014. 10.1128/AEM.00242-14 24610844 PMC4018903

[B64] West P. A. Hewitt J. H. Murphy O. M. (1979). Influence of methods of collection and storage on the bacteriology of human milk. *J. Appl. Bacteriol.* 46 269–277. 10.1111/j.1365-2672.1979.tb00820.x 572360

[B65] Whiteley A. S. Jenkins S. Waite I. Kresoje N. Payne H. Mullan B. (2012). Microbial 16S rRNA Ion Tag and community metagenome sequencing using the Ion Torrent (PGM) Platform. *J. Microbiol. Methods* 91 80–88. 10.1016/j.mimet.2012.07.008 22849830

[B66] Wylie K. M. (2017). The virome of the human respiratory tract. *Clin. Chest. Med.* 38 11–19. 10.1016/j.ccm.2016.11.001 28159153 PMC7115714

[B67] Xuan C. Shamonki J. M. Chung A. Dinome M. L. Chung M. Sieling P. A. (2014). Microbial dysbiosis is associated with human breast cancer. *PLoS One* 9:e83744. 10.1371/journal.pone.0083744 24421902 PMC3885448

[B68] Yang I. Corwin E. J. Brennan P. A. Jordan S. Murphy J. R. Dunlop A. (2016). The infant microbiome: implications for infant health and neurocognitive development. *Nurs. Res.* 65 76–88. 10.1097/NNR.0000000000000133 26657483 PMC4681407

[B69] Yolken R. H. Jones-Brando L. Dunigan D. D. Kannan G. Dickerson F. Severance E. (2014). Chlorovirus ATCV-1 is part of the human oropharyngeal virome and is associated with changes in cognitive functions in humans and mice. *Proc. Natl. Acad. Sci. U.S.A.* 111 16106–16111. 10.1073/pnas.1418895111 25349393 PMC4234575

