# Supplementary figures and images for: Shared and Distinct Features of Human Milk and Infant Stool Viromes
Source: Front Microbiol. 2018 Jun 1;9:1162. doi: 10.3389/fmicb.2018.01162 (PMC5992295; doi:10.3389/fmicb.2018.01162)

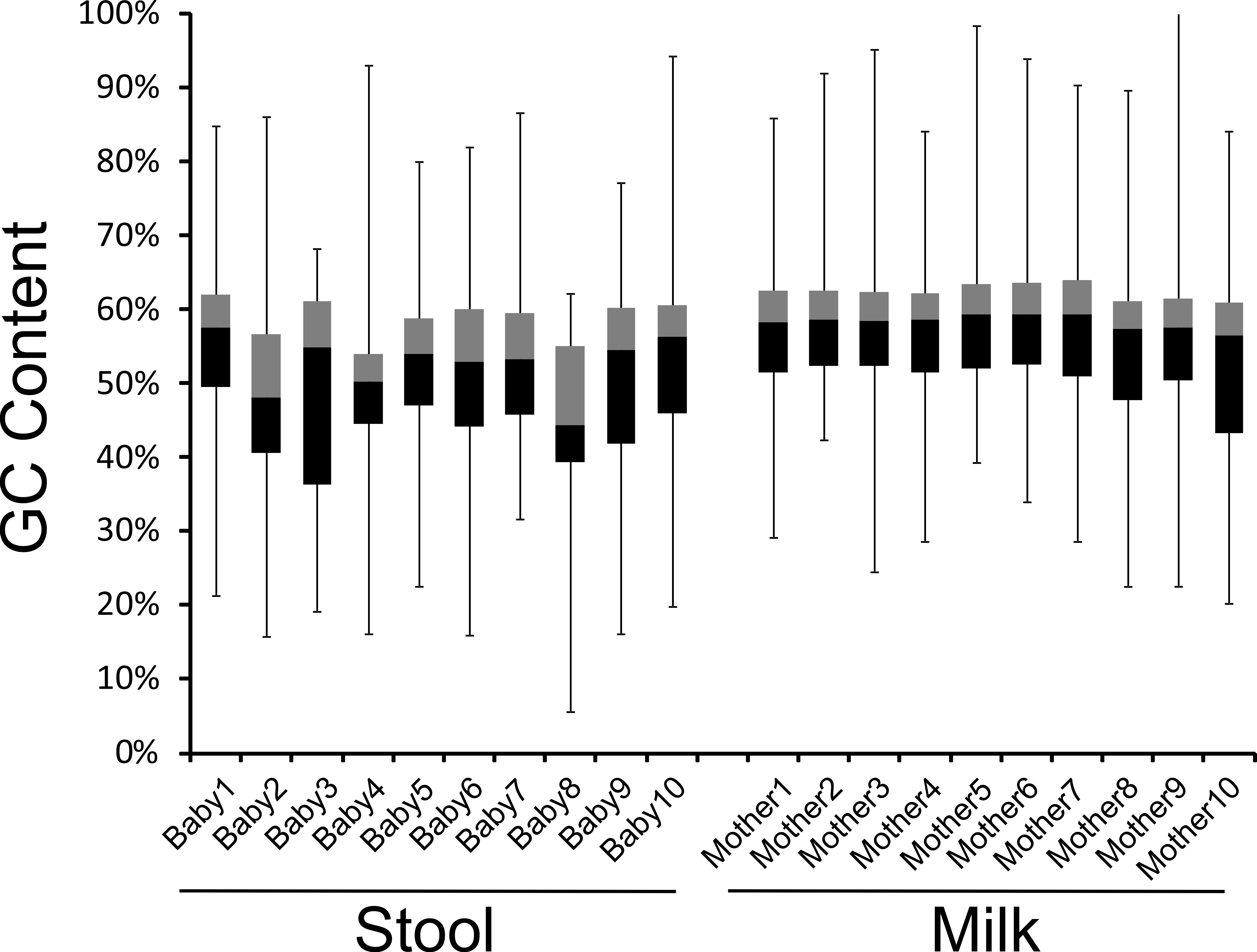

Supplement: FIGURE S1 — Box and whiskers plots demonstrating the percent G + C content amongst the virome reads recovered from infant stool (left) and human milk (right). The y-axis represents the percent G + C content. [file Image_1.TIF]

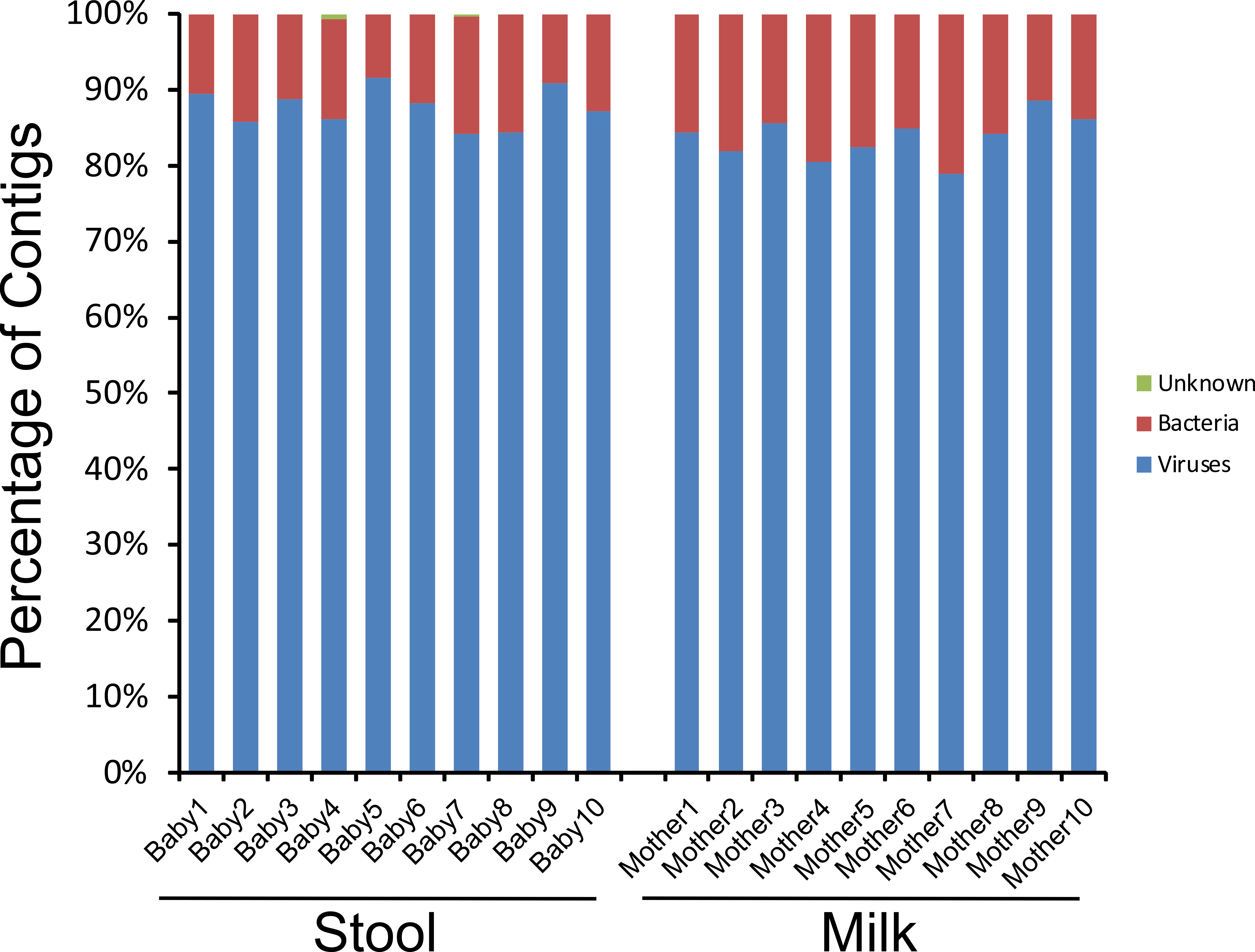

Supplement: FIGURE S2 — Bar graphs representing the proportion of contigs from infant stool and human milk with BLASTX hits to known viruses, bacteria, or without BLASTX hits. The y-axis represents the percentage of total contigs and the x-axis represents each infant stool (left) and milk (right) specimen. [file Image_2.TIF]

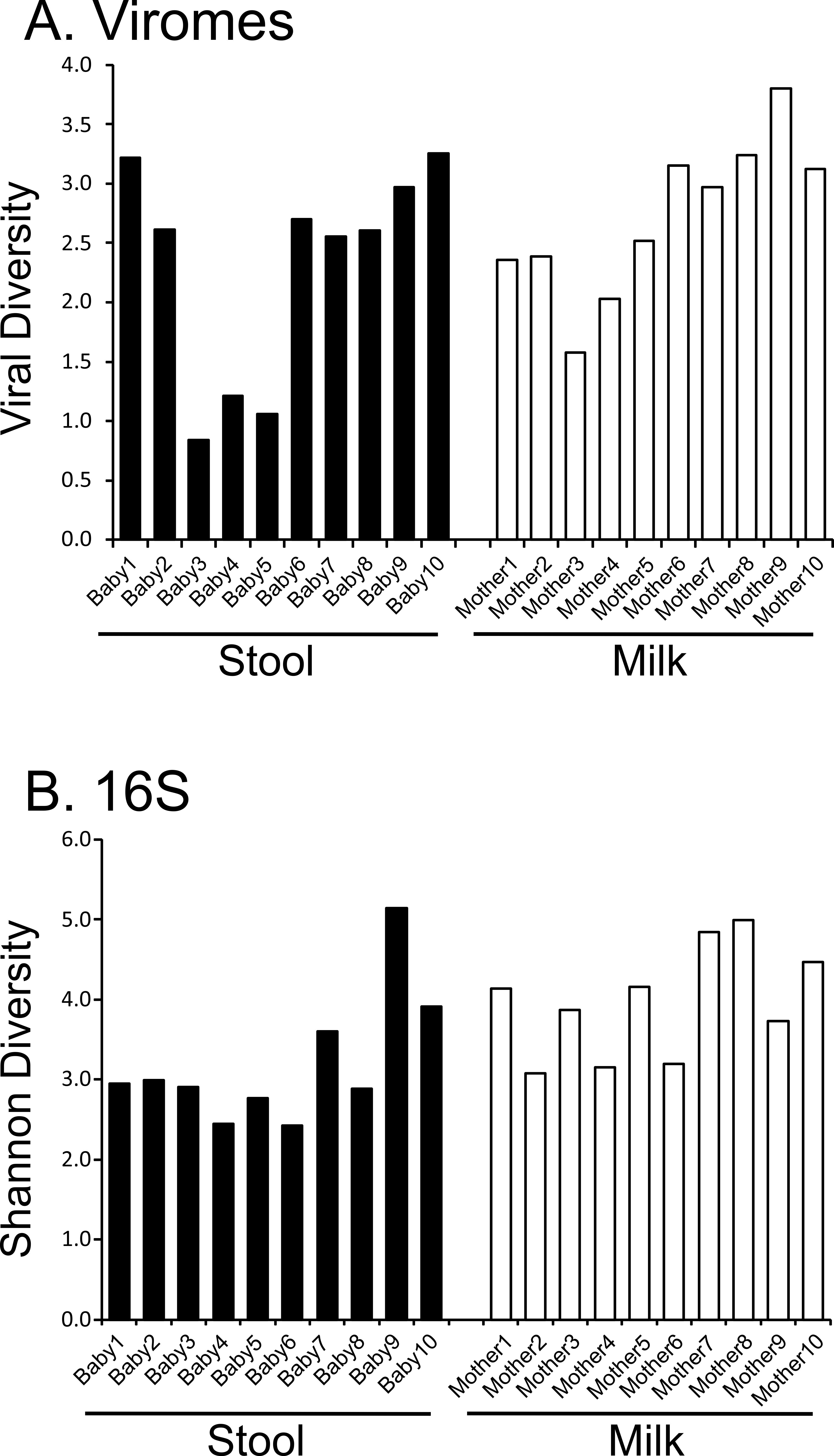

Supplement: FIGURE S3 — Bar graphs representing Shannon diversity and Homologous viral diversity in infant stool and human milk. (A) Homologous viral diversity index based on virome contigs for all infants and mothers, (B) Shannon diversity based on 16S rRNA for all infants and mothers. The x-axis represents milk (right) or infant stool (left), and the y-axis represents Shannon diversity or homologous viral diversity. [file Image_3.TIF]

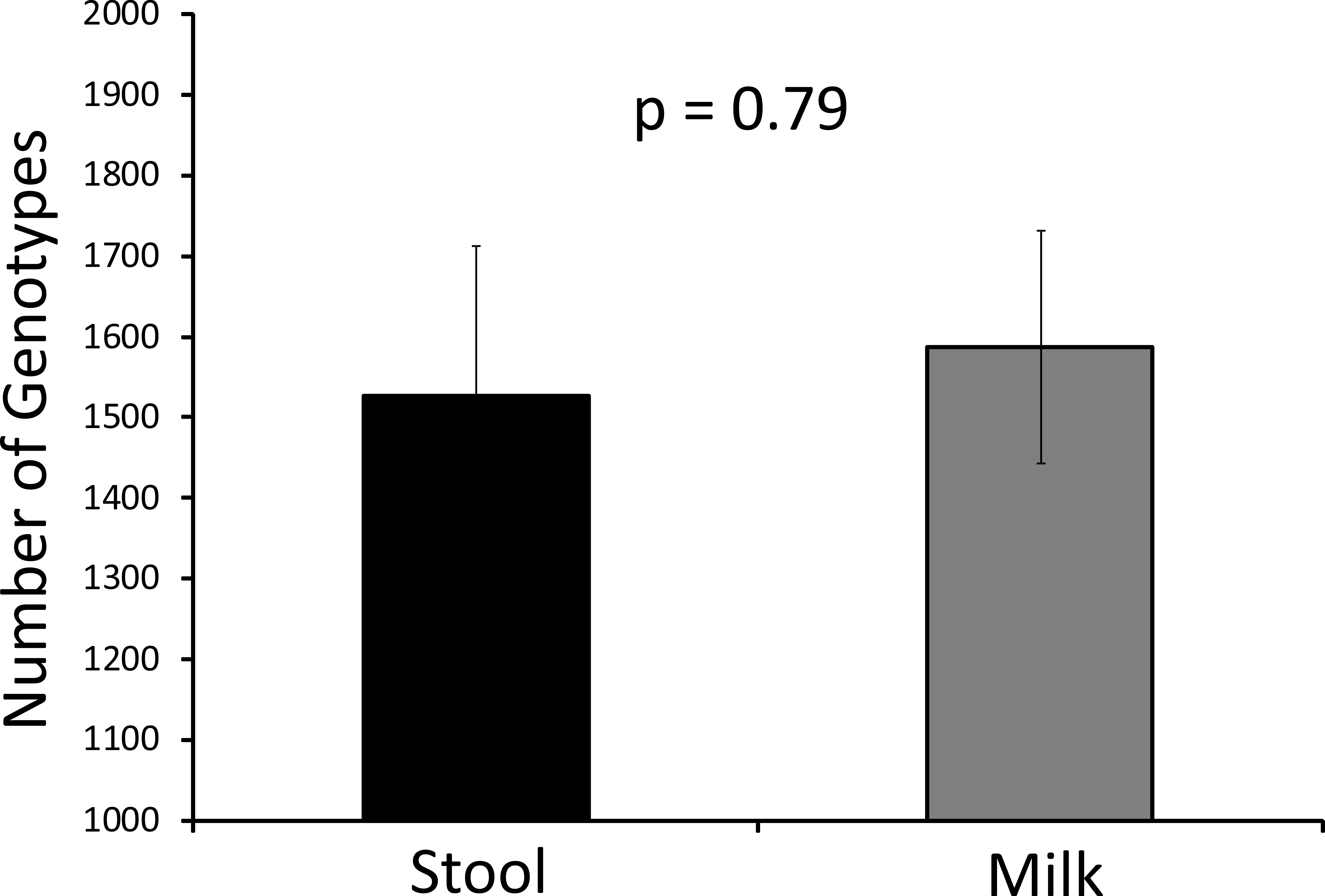

Supplement: FIGURE S4 — Bar graphs (± standard error) representing the estimated number of virus genotypes identified in infant stool and human milk. The x-axis represents milk or infant stool, and the y-axis represents estimated virus genotypes determined by the homologous virus diversity index. The p-value is shown above. [file Image_4.TIF]

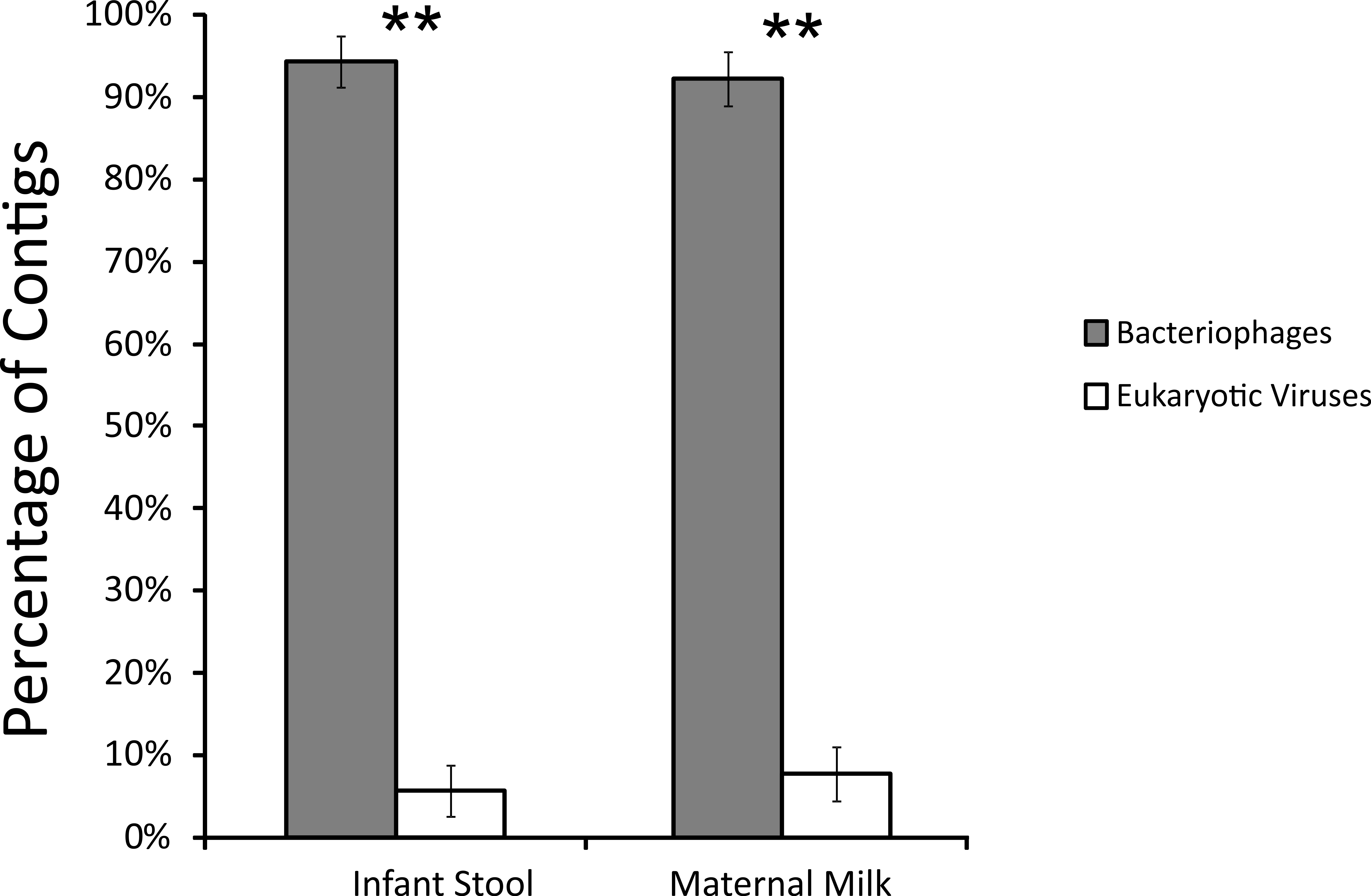

Supplement: FIGURE S5 — Bar graphs (± standard error) representing the proportion of contigs from infant stool and human milk with TBLASTX homology to bacteriophage and eukaryotic virus families. The percentage of contigs is represented on the y-axis, and the infant stool and milk specimens are shown on the x-axis. p-values ≤0.001 are represented by “∗∗”. [file Image_5.TIF]

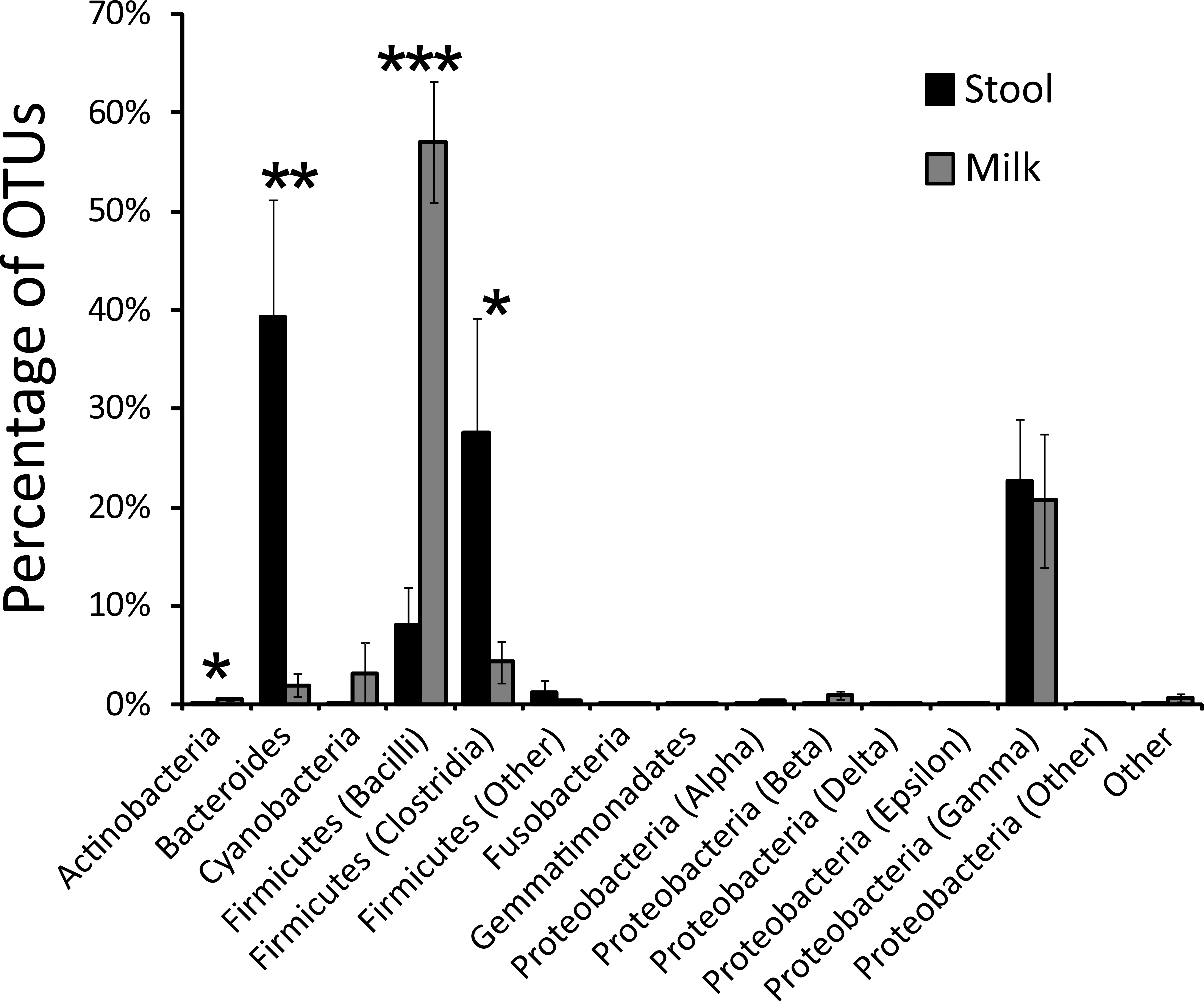

Supplement: FIGURE S6 — Bar graphs (± standard error) demonstrating the proportion of OTUs assigned to specific bacteria Classes in infant stool (black bars) and human milk (gray bars). The y-axis represents the percentage of OTUs assigned to each Class, and the x-axis represent the different bacteria Classes. The “∗” represents values that are statistically significant with p-values ≤0.01, the “∗∗” represents p-values ≤0.001, and the “∗∗∗” represents p-values ≤0.0001. The Firmicutes from the Bacilli Class belong to the Genus Streptococcus, and the Firmicutes from the Clostridia Class belong to the Genus Veillonella. [file Image_6.TIF]

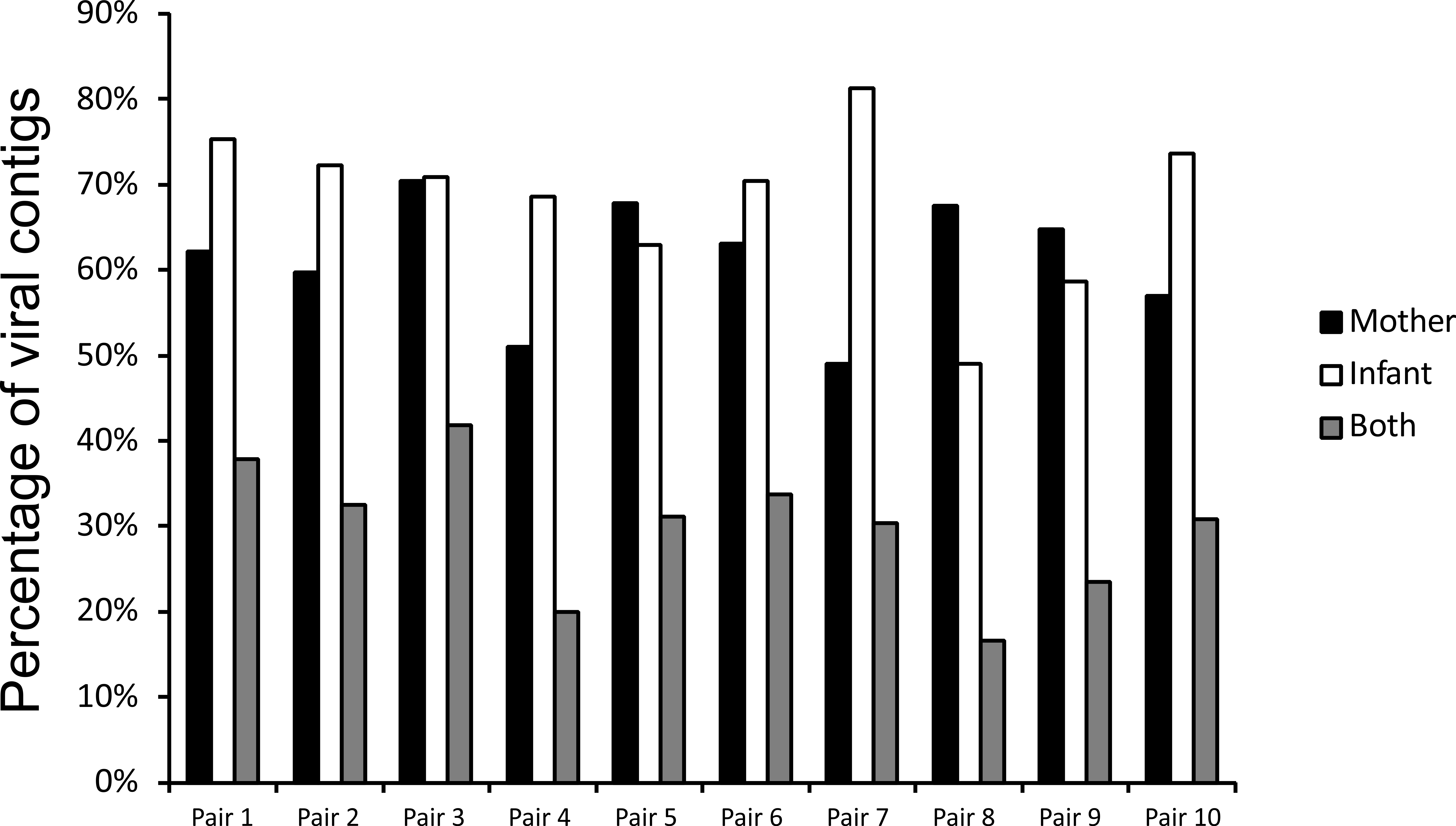

Supplement: FIGURE S7 — Bar graphs representing the relative proportion of viral contigs in each mother-infant pair assembled that include contributions from the human milk, contributions from the infant stool, or contributions from both milk and stool. The y-axis represents the percentage of viral contigs assembled, and the x-axis represents the contribution to viral contigs from the mother (black), infant (white), or both (gray). [file Image_7.TIF]

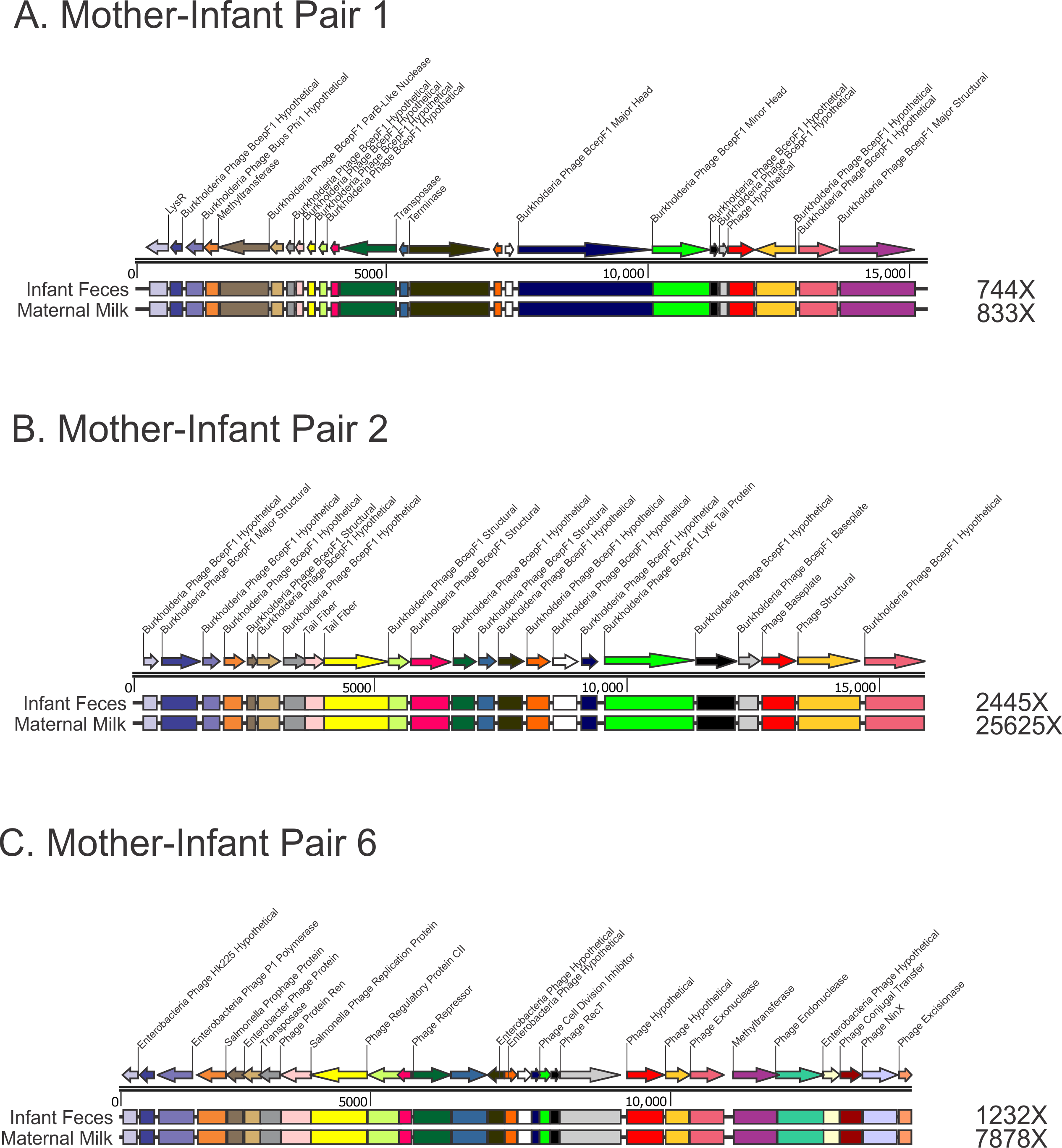

Supplement: FIGURE S8 — Assemblies of viral contigs from select mother-infant pairs. (A) represents a 15,330 nt phage from mother-infant Pair #1, (B) represents a 16,602 phage from mother-infant Pair #2, and (C) represents a 14,064 phage from mother-infant Pair #6. The average coverage from the infant stool and the human milk is shown on the right of each panel. The portions of the contig identified in the milk and stool are represented by the colored boxes. Putative ORFs and their directions are represented by the arrows and their annotations are represented in each panel. The length of the contig is denoted at the top. [file Image_8.TIF]
