# Supplementary material for: Shared and Distinct Features of Human Milk and Infant Stool Viromes
Source: Front Microbiol. 2018 Jun 1;9:1162. doi: 10.3389/fmicb.2018.01162 (PMC5992295; doi:10.3389/fmicb.2018.01162)
Supplement: TABLE S1 — Characteristics of study participants at time of specimen collection. [file Table_1.DOCX]

**Table S1. Characteristics of study participants at time of specimen collection**

|  | **Subject** | **Study Designation** | **Age** | **Sex** | **Ethnicity** | **Parity** | **Delivery Type** | **Baby weight (kg) / Maternal Pre-pregnancy BMI** | **Antibiotics** | **Current Diet** | **Exclusive Breastfeeding^2^** |
| --- | --- | --- | --- | --- | --- | --- | --- | --- | --- | --- | --- |
| Pair 1 | | | | | | | | | | | |
|  | Baby1 | BB1 | 9 days | M | Hispanic | Singleton | C-section | 3.3 | No | Mix | No |
|  | Mother1 | M131 | 19 years | F | Hispanic | 2 | N/A | 29.5 | Yes^1^ | N/A | N/A |
| Pair 2 | | | | | | | | | | | |
|  | Baby2 | BB2 | 8 days | F | Hispanic | Singleton | Vaginal | 3.6 | No | BF^3^ | Yes |
|  | Mother2 | M138 | 24 years | F | Hispanic | 4 | N/A | 33.3 | Yes^1^ | N/A | N/A |
| Pair 3 | | | | | | | | | | | |
|  | Baby3 | BB3 | 6 days | M | Hispanic | Singleton | Vaginal | 2.8 | No | BF^3^ | No |
|  | Mother3 | M132 | 33 years | F | Hispanic | 4 | N/A | 19.5 | No | N/A | N/A |
| Pair 4 | | | | | | | | | | | |
|  | Baby4 | BB4 | 7 days | F | Hispanic | Singleton | Vaginal | 3.2 | No | Mix | No |
|  | Mother4 | M136 | 20 years | F | Hispanic | 1 | N/A | 23.4 | No | N/A | N/A |
| Pair 5 | | | | | | | | | | | |
|  | Baby5 | BB8 | 10 days | M | Hispanic | Singleton | C-section | 4.3 | No | Mix | No |
|  | Mother5 | M113 | 29 years | F | Hispanic | 2 | N/A | 31.2 | Yes^1^ | N/A | N/A |
| Pair 6 | | | | | | | | | | | |
|  | Baby6 | BB9 | 9 days | F | Hispanic | Singleton | C-section | 4.4 | No | BF^3^ | Yes |
|  | Mother6 | M118 | 36 years | F | Hispanic | 4 | N/A | Unknown | Yes^1^ | N/A | N/A |
| Pair 7 | | | | | | | | | | | |
|  | Baby7 | BB10 | 7 days | M | Hispanic | Singleton | Vaginal | 3.2 | No | Mix | No |
|  | Mother7 | M141 | 25 years | F | Hispanic | 1 | N/A | 31.2 | No | N/A | N/A |
| Pair 8 | | | | | | | | | | | |
|  | Baby8 | BB11 | 4 days | M | Hispanic | Singleton | Vaginal | 3.6 | No | Mix | No |
|  | Mother8 | M112 | 20 years | F | Hispanic | 1 | N/A | 18.9 | No | N/A | N/A |
| Pair 9 | | | | | | | | | | | |
|  | Baby9 | BB12 | 7 days | M | Hispanic | Singleton | Vaginal | 3.5 | No | Mix | No |
|  | Mother9 | M128 | 26 years | F | Hispanic | 1 | N/A | 26.4 | No | N/A | N/A |
| Pair 10 | | | | | | | | | | | |
|  | Baby10 | BB13 | 7 days | M | Hispanic | Singleton | Vaginal | 3.7 | No | BF^3^ | Yes |
|  | Mother10 | M134 | 38 years | F | Hispanic | 4 | N/A | 21.4 | No | N/A | N/A |

^1^ Received intrapartum antibiotics

^2^ Defined as never received formula

^3^ Breastfed
