# Supplementary material for: Shared and Distinct Features of Human Milk and Infant Stool Viromes
Source: Front Microbiol. 2018 Jun 1;9:1162. doi: 10.3389/fmicb.2018.01162 (PMC5992295; doi:10.3389/fmicb.2018.01162)
Supplement: TABLE S2 — Contig metrics from milk and stool. [file Table_2.DOCX]

**Supplemental Table 2: Contig metrics from milk and stool**

|  |  | Designation | Reads | Contigs | Mean Length | Max Length | N50 | Percent In Contigs^a^ |
| --- | --- | --- | --- | --- | --- | --- | --- | --- |
| Milk |  |  |  |  |  |  |  |  |
|  | Mother 1 | M131 | 7,088,452 | 1,329 | 1,038 | 14,027 | 1,752 | 93.0% |
|  | Mother 2 | M138 | 5,829,918 | 1,343 | 956 | 21,070 | 1,524 | 94.1% |
|  | Mother 3 | M132 | 8,800,692 | 1,213 | 1,052 | 25,582 | 1,854 | 74.1% |
|  | Mother 4 | M136 | 6,053,886 | 1,231 | 1,015 | 37,132 | 1,628 | 93.7% |
|  | Mother 5 | M113 | 8,897,335 | 1,760 | 1,051 | 50,753 | 1,914 | 92.2% |
|  | Mother 6 | M118 | 7,529,895 | 1,944 | 1,034 | 21,431 | 1,718 | 93.9% |
|  | Mother 7 | M141 | 9,343,466 | 1,954 | 970 | 27,110 | 1,677 | 92.5% |
|  | Mother 8 | M112 | 9,162,985 | 2,405 | 1,206 | 15,094 | 2,384 | 93.6% |
|  | Mother 9 | M128 | 11,348,259 | 2,842 | 1,103 | 21,856 | 1,998 | 92.8% |
|  | Mother 10 | M134 | 13,572,808 | 2,275 | 1,056 | 19,298 | 1,897 | 91.8% |
| Stool |  |  |  |  |  |  |  |  |
|  | Baby 1 | BB1 | 8,329,926 | 1,825 | 1,078 | 28,316 | 1,908 | 93.0% |
|  | Baby 2 | BB2 | 13,435,230 | 2,486 | 913 | 32,620 | 1,577 | 75.2% |
|  | Baby 3 | BB3 | 17,693,106 | 2,133 | 994 | 27,167 | 1,716 | 93.8% |
|  | Baby 4 | BB4 | 12,631,540 | 2,291 | 1,132 | 41,295 | 2,246 | 94.3% |
|  | Baby 5 | BB8 | 9,070,942 | 994 | 1,201 | 27,394 | 2,593 | 96.4% |
|  | Baby 6 | BB9 | 9,761,513 | 1,624 | 1,133 | 34,570 | 1,954 | 95.9% |
|  | Baby 7 | BB10 | 11,401,642 | 2,747 | 1,048 | 16,928 | 1,795 | 94.8% |
|  | Baby 8 | BB11 | 10,867,411 | 1,811 | 1,084 | 24,242 | 1,974 | 93.7% |
|  | Baby 9 | BB12 | 10,624,494 | 2,174 | 1,204 | 32,300 | 2,505 | 92.7% |
|  | Baby 10 | BB13 | 10,697,481 | 2,844 | 1,094 | 20,693 | 1,934 | 92.9% |

^a^Percentage of reads that were assembled into contigs
